# OR2H2 Activates CAMKKβ–AMPK–Autophagy Signaling Axis and Suppresses Senescence in VK2/E6E7 Cells

**DOI:** 10.3390/ph16091221

**Published:** 2023-08-29

**Authors:** Ji Min Kim, Sina Dziobaka, Ye Eun Yoon, Ha Lim Lee, Ji Hyun Jeong, In-Ryeong Lee, Daniel Weidinger, Changwon Yang, Deokho Kim, Yalcin Gulperi, Cheol-Koo Lee, Jeongwon Sohn, Gwonhwa Song, Hanns Hatt, Sung-Joon Lee

**Affiliations:** 1 Department of Biotechnology, School of Life Science and Biotechnology for BK21 PLUS, Korea University, Seoul 02855, Republic of Korea; jamiekim19@gmail.com (J.M.K.); yeeun916@korea.ac.kr (Y.E.Y.); hagurim94@naver.com (H.L.L.); embroider13@naver.com (J.H.J.); lee3231817@naver.com (I.-R.L.); ycw117@korea.ac.kr (C.Y.); mzxcmz@naver.com (D.K.); gulperiyalcin@korea.ac.kr (Y.G.); cklee2005@korea.ac.kr (C.-K.L.);; 2 Department of Cell Physiology, Ruhr-University Bochum, 44791 Bochum, Germany; sina.dziobaka@rub.de (S.D.); daniel.weidinger@rub.de (D.W.); 3 Division of Biotechnology, College of Life Sciences and Biotechnology, Korea University, Seoul 02855, Republic of Korea; 4 Department of Biochemistry and Molecular Biology, Korea University College of Medicine, Seoul 02842, Republic of Korea; biojs@korea.ac.kr; 5 Korea Institute of Molecular Medicine and Nutrition, Seoul 02842, Republic of Korea; 6 Department of Food Bioscience and Technology, College of Life Sciences and Biotechnology, Korea University, Seoul 02855, Republic of Korea; 7 Interdisciplinary Program in Precision Public Health, Korea University, Seoul 02846, Republic of Korea; 8 BK21 Four Institute of Precision Public Health, Korea University, Seoul 02846, Republic of Korea

**Keywords:** olfactory receptor, autophagy, apoptosis, senescence

## Abstract

Olfactory receptors are expressed in multiple extra-nasal tissues and these ectopic olfactory receptors mediate tissue-specific functions and regulate cellular physiology. Ectopic olfactory receptors may play key roles in tissues constantly exposed to odorants, thus the functionality of these receptors in genital tissues is of particular interest. The functionality of ectopic olfactory receptors expressed in VK2/E6E7 human vaginal epithelial cells was investigated. OR2H2 was the most highly expressed olfactory receptor expressed in VK2/E6E7 cells, and activation of OR2H2 by aldehyde 13-13, a ligand of OR2H2, increased the intracellular calcium and cAMP concentrations. Immunoblotting demonstrated that activation of OR2H2 by aldehyde 13-13 stimulated the CAMKKβ–AMPK–mTORC1–autophagy signaling axis, and that these effects were negated by OR2H2 knockdown. AMPK is known to regulate senescence; consequently, we investigated further the effect of aldehyde 13-13 on senescence. In H_2_O_2_-induced senescent cells, activation of OR2H2 by aldehyde 13-13 restored proliferation, and reduced the expression of senescence markers, P16 and P19. Additionally, aldehyde 13-13 induced apoptosis of H_2_O_2_-induced senescent cells, compared with non-senescent normal cells. In vivo, aldehyde 13-13 increased the lifespan of Caenorhabditis elegans and budding yeast. These findings demonstrate that OR2H2 is a functional receptor in VK2/E6E7 cells, and that activation of OR2H2 activates the AMPK–autophagy axis, and suppresses cellular aging and senescence, which may increase cellular health.

## 1. Introduction

Olfactory receptors (ORs), part of the G protein-coupled receptor (GPCR) superfamily, are primarily expressed in the cilia of olfactory sensory neurons where olfactory perception is initiated upon odorant binding to ORs. These receptors are also ectopically expressed in almost all tissues including liver, lung, sperm, colon, and epithelial cells [1,2]. These ectopic expressions of ORs underscore their regulatory roles in biological and physiological processes, such as sperm chemotaxis, energy metabolism, wound healing, and hair growth [3,4,5,6]. Currently, only a limited number of ligands for ORs have been identified [7], and a mere 20–30% of human ORs have been deorphanized and finding a potent ligand for human OR remains a challenge. OR2H2 is one such deorphanized human olfactory receptor [8], with aldehyde 13-13 as its ligand. OR2H2, ectopically expressed in human myoblasts, is activated by aldehyde 13-13 and influences skeletal muscle differentiation by inhibiting myoblast fusion [9].

ORs are also present in human genital organs. GTEx analysis (https://gtexportal.org) indicates that OR2H2 is found in vaginal tissue, though its exact function remains elusive. The human vaginal epithelium is a multi-layered, non-keratinized squamous epithelium without glands that envelops the inner surface of the vagina.

Impaired function of the vaginal epithelium, especially in older women, can compromise vaginal health, increasing the risk of vaginal atrophy due to thinning of the vagina’s inner layer [10]. Both autophagy function and senescence progression are instrumental in the onset of vaginal atrophy. Many studies have delved into the induction of autophagy in human genital cells to preserve vaginal health. For instance, Shao et al. discussed how the Chinese traditional herb prescription JieZe-1 enhanced autophagy by inhibiting the PI3K/Akt/mTOR signaling axis in VK2/E6E7 cells [11]. Yang et al. demonstrated that inducing autophagy in VK2/E6E7 cells bolstered the barrier function of the vaginal epithelium [12,13]. A decline in autophagy activity is often linked to senescence. As autophagy facilitates the removal of impaired cellular components, it can prevent the accumulation of cellular damage leading to senescence, making it a potential protective mechanism against premature aging.

AMP-activated protein kinase (AMPK), a central regulator of energy metabolism, stands among the regulators that induce autophagy, hence promoting cellular homeostasis and senescence protection. AMPK propels autophagy through various means. For example, AMPK triggers inhibitory phosphorylation on TSC1/2 and Raptor, which inhibit autophagy and phosphorylates ULK1, initiating autophagy [14]. Concurrently, AMPK activation is known to curb cellular senescence via several pathways. AMPK-dependent phosphorylation of p27^kip1^ enhances p27^kip1^ stability, which in turn reduces cell-senescence indicators, such as beta-galactosidase, P16^INK4A^, and P21 [15]. Furthermore, AMPK can inhibit senescence by activating sirtuin 1 (SIRT1) [16], an anti-aging protein entangled in numerous signaling routes, including cell senescence [17]. 

In this study, we explored the function of OR2H2, the most predominant OR in VK2/E6E7 cells. Results from phosphokinase array experiments revealed that aldehyde 13-13 robustly initiated AMPK phosphorylation. Consequently, we postulated that OR2H2 might bolster cellular health through the regulation of autophagy and senescence in VK2/E6E7 cells.

In our findings, we highlight the functionality of ectopic OR expression in VK2/E6E7 human vaginal epithelial cells. OR2H2 was profusely expressed in VK2/E6E7 cells, and its activation by its ligand, aldehyde 13-13, initiated intracellular calcium signaling and CAMKKβ–AMPK–autophagy pathways. Activating OR2H2 revitalized cell proliferation and curtailed primary markers of cellular senescence in H_2_O_2_-induced senescent cells. OR2H2 activation also exhibited a senolytic effect by inducing apoptosis in these cells. In sum, our findings suggest that OR2H2 activation augments the autophagy pathway and impedes cellular aging via AMPK activation, proposing OR2H2 as a potential molecular target to enhance cellular health and combat aging. 

## 2. Results

### 2.1. Aldehyde 13-13 Increases the Intracellular Ca^2+^ and cAMP Levels in VK2/E6E7 Cells

**Figure 1:**
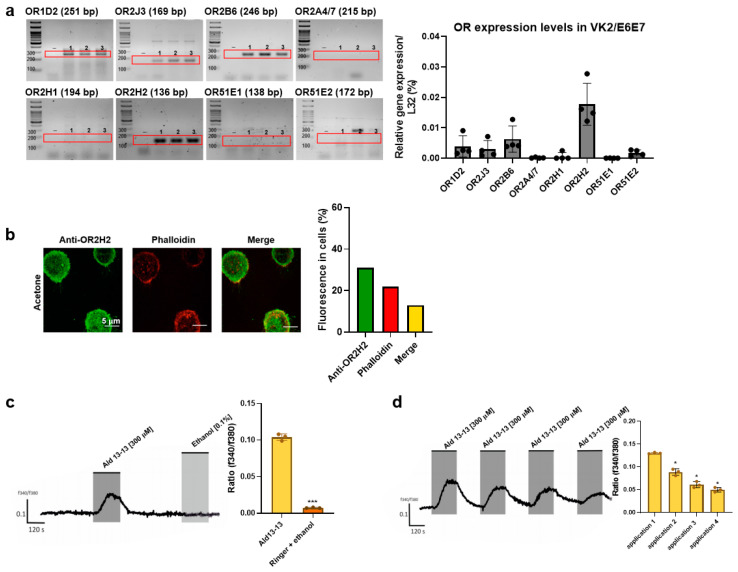
OR2H2-mediated increase in intracellular Ca^2+^ level in VK2/E6E7 cells: (**a**) Expression of OR2H2 and other ORs in VK2/E6E7 cells determined by RT-PCR. “-” represents a negative control without reverse transcriptase during cDNA synthesis. Experiments were conducted in triplicates, denoted as 1, 2, and 3 in the gel images. (**b**) OR2H2 expression in VK2/E6E7 cells observed through immunocytochemical staining, using anti-OR2H2 antibody (green). Actin was stained with phalloidin (red). Scale bars represent 5 µm. Fluorescence quantification is shown in the right panel. (**c**) Intracellular Ca^2+^ concentration after aldehyde 13-13 (300 µM) stimulation in VK2/E6E7 cells. The application time (120 s) is indicated by the grey square. Solvent ethanol (0.1%) was applied to assess potential solvent-mediated reactions. (**d**) Verification of Ca^2+^ desensitization with repeated aldehyde 13-13 (300 µM) stimulation. Cells were repetitively stimulated with aldehyde 13-13 (300 µM) for two minutes, while control cells received a vehicle treatment (ethanol in Ringer’s solution) repeatedly for the same duration. Ratiometric calcium imaging and normalized mean values from all ratio graphs are presented. Experiments were carried out in either three or four replicates. Ald 13-13 signifies aldehyde 13-13. Two-tailed unpaired *t*-tests were employed for either multiple or two-group comparisons. Significance markers: *, *p* < 0.05; ***, *p* < 0.001.

**Figure 2:**
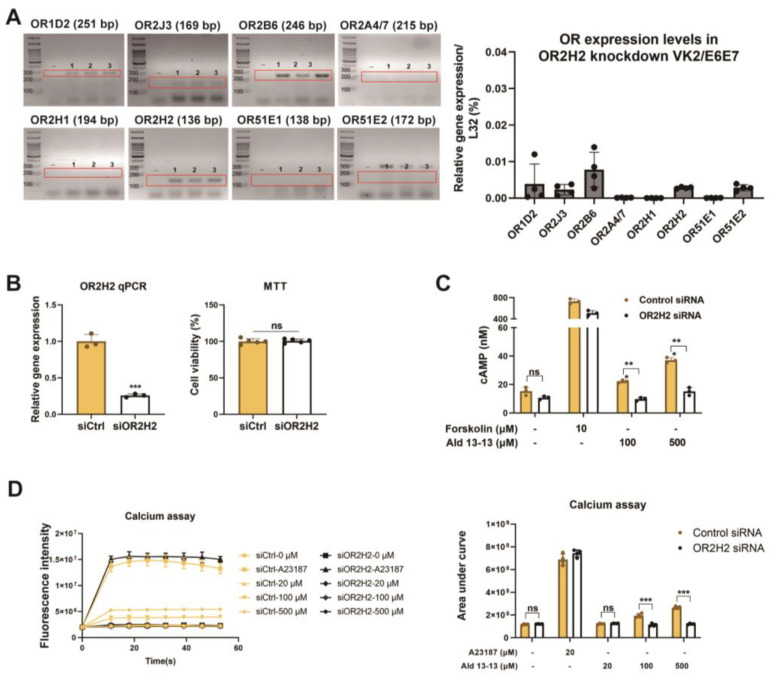
Aldehyde 13-13 increases intracellular Ca^2+^ and cAMP levels in VK2/E6E7 cells, but not in OR2H2 knockdown VK2/E6E7 cells: (**A**) OR expressions in VK2/E6E7 cells post-OR2H2 gene knock-down via OR2H2 siRNA. “-” is a negative control without reverse transcriptase during cDNA synthesis. Experiments were performed in triplicates, as indicated 1, 2, and 3 in the gel images. (**B**) OR2H2 expression in VK2/E6E7 cells measured by qPCR (left panel) and cell viability evaluated by MTT assays (right panel) post siRNA transfection. (**C**) Intracellular cAMP and (**D**) Ca^2+^ concentration post-aldehyde 13-13 (100, 500, 1000 µM) stimulation in control siRNA-treated VK2/E6E7 cells and OR2H2 siRNA-treated VK2/E6E7 cells. Experiments were conducted in three to five replicates. Ald 13-13 denotes aldehyde 13-13. Forskolin represents a cAMP agonist; A23187 is a Ca^2+^ agonist. Two-tailed unpaired *t*-tests were used for multiple or two-group comparisons. Significance markers: *, *p* < 0.05; **, *p* < 0.01; ***, *p* < 0.001.

We began by examining ectopic OR expression in VK2/E6E7 cells. Based on GTEx analysis (https://gtexportal.org), ORs were chosen, and their expression was validated via RT-PCR and qPCR. OR1D2, OR2J3, OR2B6, and OR2H2 were expressed in VK2/E6E7 cells (Figure 1a). Among these, OR2H2 displayed the most significant expression. Immunocytochemistry illustrated that OR2H2 was localized to the cell membrane and the perinuclear region of the cytosol (Figure 1b). Approximately 13% of the OR2H2 signal overlapped with the phalloidin signal, indicating a significant presence of OR2H2 protein in the plasma membrane (Figure 1b, right panel). In second messenger assays, aldehyde 13-13 (a ligand for OR2H2) notably boosted intracellular calcium levels, unlike propionic acid (a ligand of OR51E2) and lactic acid (a ligand of Olfr78) (Figure 1c,d and Figure S1). We subsequently delved deeper into the functionality of OR2H2 in VK2/E6E7 cells. Confirming earlier findings, aldehyde 13-13 instigated a robust intracellular Ca^2+^ response in VK2/E6E7 cells (Figure 1c). When VK2/E6E7 cells were continuously exposed to aldehyde 13-13 (300 µM), there was a consistent decline in Ca^2+^ levels, hinting at desensitization of the OR2H2–calcium signaling pathway (Figure 1d). We then explored whether the second messengers generated by aldehyde 13-13 were dependent on OR2H2. In cells where the OR2H2 gene was knocked down using OR2H2 siRNA, OR2H2 expression diminished by approximately 80% without affecting other ORs shown in Figure 1a. The OR2H2 siRNA left VK2/E6E7 cell viability unchanged (Figure 2A,B). While aldehyde 13-13 significantly upregulated intracellular cAMP and Ca^2+^ concentrations, these effects were negated in cells with OR2H2 knocked down (Figure 2C,D). Collectively, these findings indicate that the impacts of aldehyde 13-13 on VK2E6E7 cells are mediated through OR2H2.

### 2.2. Aldehyde 13-13 Increased the Intracellular Ca^2+^ Level by Activating Multiple Calcium Channels

**Figure 3:**
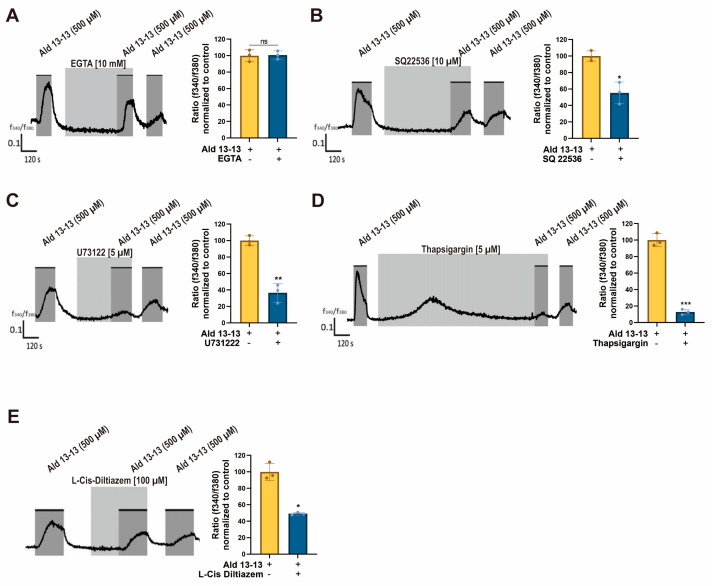
Aldehyde 13-13-driven intracellular Ca^2+^ induction is counteracted by Ca^2+^ channel inhibitors in VK2/E6E7 cells: (**A**) Intracellular Ca^2+^ concentration with aldehyde 13-13 (500 µM) stimulation under Ca^2+^-free conditions. (**B**–**E**) Intracellular Ca^2+^ concentrations after combined treatment with aldehyde 13-13 (500 µM) and the Ca^2+^ inhibitors SQ22536 (10 µM), U73122 (5 µM), thapsigargin (5 µM), and L-Cis diltiazem (100 µM). Experiments were executed as outlined in the Experimental Procedure. Ratiometric calcium imaging and normalized means of ratios are displayed. Experiments were performed in three replicates. Ald 13-13 represents aldehyde 13-13. Two-tailed unpaired *t*-tests were utilized for either multiple or two-group comparisons. Significance markers: *, *p* < 0.05; **, *p* < 0.01; ***, *p* < 0.001.

Intracellular Ca^2+^ levels can be elevated either by influx from external sources or by releasing stored intracellular Ca^2+^ from organelles such as the endoplasmic reticulum or mitochondria. To discern the origin of the Ca^2+^ surge following aldehyde 13-13 treatment, we conducted a calcium imaging test under Ca^2+^-free conditions. The ratiometric calcium imaging revealed no amplitude change during these calcium-free conditions. This suggests that the rise in intracellular Ca^2+^ levels due to aldehyde 13-13 is not reliant on extracellular calcium but potentially originates from the internal calcium reservoir (Figure 3A). To pinpoint this source, VK2/E6E7 cells were simultaneously treated with Ca^2+^ inhibitors and aldehyde 13-13. The Ca^2+^ boost instigated by aldehyde 13-13 diminished in cells co-treated with an adenylyl cyclase III inhibitor (SQ22536), a PLC inhibitor (U73122), a SERCA inhibitor (thapsigargin), and a CNG channel inhibitor (L-cis diltiazem) (Figure 3B–E). Thus, aldehyde 13-13 amplifies intracellular Ca^2+^ concentrations in VK2/E6E7 cells by engaging multiple Ca^2+^ channels. 

### 2.3. Activation of OR2H2 Induces the Calcium–CaMKKβ–AMPK Signaling Pathway

**Figure 4:**
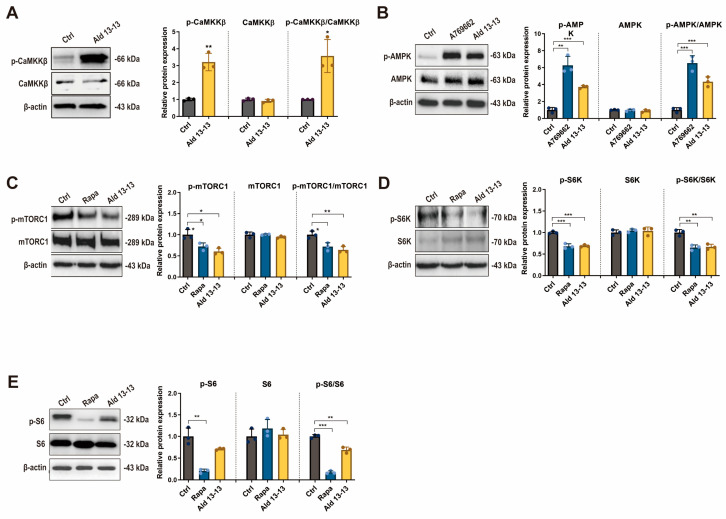
OR2H2 activation triggers the CaMKKß–AMPK–mTORC1 signaling pathway in VK2/E6E7 cells: (**A**) Immunoblot results for phospho-CaMKK-ß, CaMKK-ß, and p-CaMKK-ß/CaMKK-ß ratio; (**B**) phospho-AMPK, AMPK, and p-AMPK/AMPK ratio; (**C**) phospho-mTORC1, mTORC1, and p-mTORC1/mTORC1 ratio; (**D**) phospho-S6K, S6K, and p-S6K/S6K ratio; (**E**) phospho-S6, S6, and p-S6/S6 ratio. Tests were conducted in triplicate. Ctrl denotes control (0.1% ethanol); A769662 (AMPK agonist), 100 µM; Ald 13-13 refers to aldehyde 13-13 at 500 µM; Rapa (rapamycin) is at 100 nM. Data are displayed as mean ± SD. Two-tailed unpaired *t*-test was used for multiple or two-group comparisons. *, *p* < 0.05; **, *p* < 0.01; ***, *p* < 0.001.

We next explored the signal transduction pathways influenced by OR2H2. In a phosphokinase array analysis evaluating the phosphorylation levels of 43 kinases, aldehyde 13-13 significantly enhanced the phosphorylation of AMPKα1 (Figure S2). Given that calcium signaling can trigger various components including CAMKKβ and considering that aldehyde 13-13 boosted AMPK phosphorylation in kinase array assays, CAMKKβ became our primary focus. CAMKKβ acts as an upstream kinase for AMPK. Upon aldehyde 13-13 stimulation, immunoblotting demonstrated the enhanced phosphorylation of both CaMKKß (an upstream kinase of AMPK) and AMPKα1, signifying OR2H2 activation (Figure 4A,B and Figure S3a,b).

AMPK plays a pivotal role in regulating cellular energy metabolism and homeostasis [18]. Its activation is considered a prospective therapeutic target for metabolic disorders [19] and aging [20]. AMPK exhibits diverse biological functions, predominantly activating the autophagy pathway through the phosphorylation inhibition of mTORC1 [21]. Given that S6K and S6 are downstream signaling components of mTORC1, their phosphorylation was also examined. Immunoblotting revealed a significant decrease in phosphorylation levels of mTORC1, S6K, and S6 (a target of S6K) in VK2/E6E7 cells post aldehyde 13-13 treatment (Figure 4C–E and Figure S3c–e). The ratios of phosphorylated-to-total mTOR, S6K, and S6 were notably diminished. Consequently, OR2H2 activation by aldehyde 13-13 stimulates the CaMKKß–AMPK–mTORC1 signaling pathway.

### 2.4. Activation of OR2H2 Induces Autophagy Pathways and Autolysosome Formation

**Figure 5:**
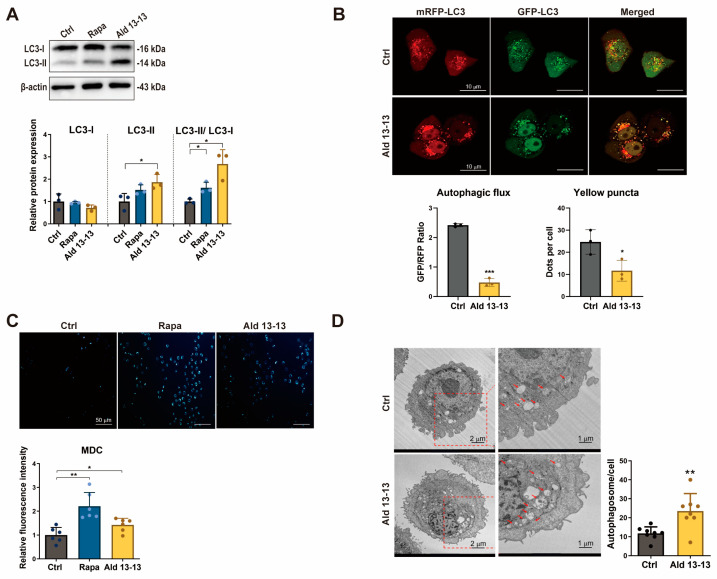
Aldehyde 13-13 transitions autophagic flux from autophagosome to autolysosome formation: (**A**) Immunoblotting for LC3I, LC3II, and LC3II/LC3I ratio; (**B**) Confocal visualization of autophagosome and autolysosome formation using mRFP-GFP-LC3 fluorescence. Bar scale: 10 µm; (**C**) Autophagy vacuole detection via MDC staining. Bar scale: 50 µm; (**D**) Transmission electron microscopy (TEM) depiction of autolysosome formation in VK2/E6E7 cells. Bar scale: 1 µm. Tests were conducted in three to eight repetitions. Ctrl, control (0.1% ethanol); Rapa (rapamycin, 100 nM); Ald 13-13 is aldehyde 13-13 at 500 µM. Data are expressed as mean ± SD. Two-tailed unpaired *t*-test was used for multiple or two-group comparisons. *, *p* < 0.05; **, *p* < 0.01; ***, *p* < 0.001.

The initiation of the AMPK–mTORC1 signaling route may induce cellular autophagy [14]. Immunoblotting demonstrated an increased LC3-II to LC3-I ratio—a primary autophagosome formation marker—in cells treated with aldehyde 13-13 (Figure 5A and Figure S4), suggesting autophagy amplification upon OR2H2 activation. We assessed further the influence of aldehyde 13-13 on autophagic flux in VK2/E6E7 cells. Post-transfection with the mRFP-mGFP-LC3 tandem fluorescence-tagged vector, autolysosome generation was observed. This tandem fluorescence-tagged LC3 allows the differentiation of acidic autolysosomes (red puncta) from neutral autophagosomes (yellow puncta) due to GFP degradation. In comparison with controls, OR2H2 activation showed augmented formation of autophagosomes and autolysosomes (Figure 5B). The elevation in autophagy vacuoles due to aldehyde 13-13 was further validated by *MDC* staining (Figure 5C). Moreover, transmission electron microscopy revealed a significant upsurge in autolysosomes post-aldehyde 13-13 treatment (Figure 5D). Thus, OR2H2 activation promotes autophagy in VK2/E6E7 cells. 

### 2.5. OR2H2-Dependent–AMPK–Autophagy Signaling Pathway Mediates Suppression of Cellular Senescence

**Figure 6:**
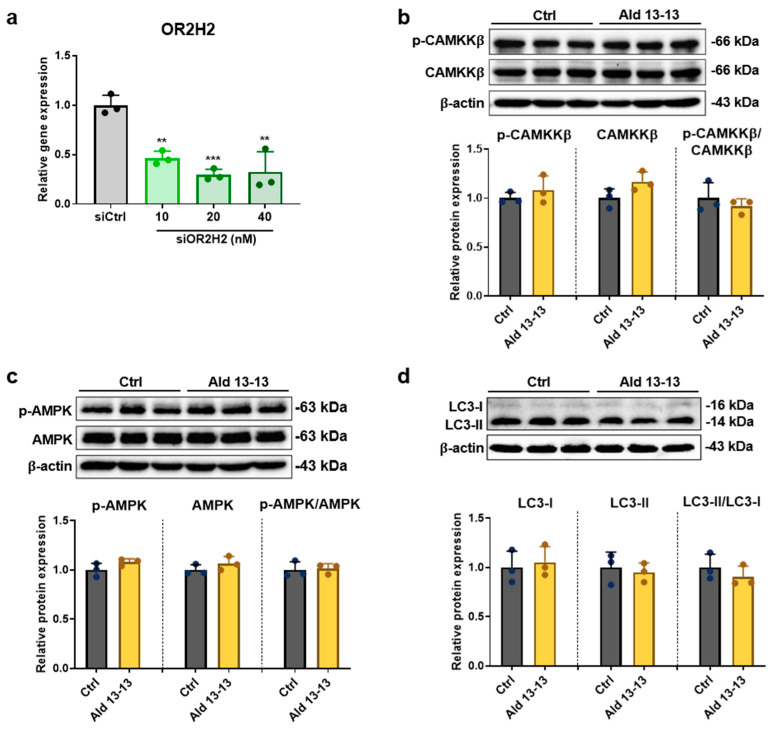
Aldehyde 13-13’s influence on the CAMKKβ–AMPK–autophagy pathway is negated following OR2H2 gene knockdown: (**a**) OR2H2 gene knockdown analysis in cells transfected with control (scrambled) and varying concentrations of OR2H2 siRNA; (**b**) Immunoblot results for phospho-CaMKK-ß, CaMKK-ß, and p-CaMKK-ß/CaMKK-ß ratio; (**c**) phospho-AMPK, AMPK, and p-AMPK/AMPK ratio; (**d**) LC3I, LC3II, and LC3II/LC3I ratio. Experiments were performed in triplicate. Ctrl, control (0.1% DMSO); Ald 13-13, aldehyde 13-13 (500 µM). Data are shown as mean ± SD. Two-tailed unpaired *t*-test was used for multiple or two-group comparisons. **, *p* < 0.01; ***, *p* < 0.001.

We undertook OR2H2 knockdown experiments to validate the dependency of the CaMKKβ–AMPK–autophagy signaling on OR2H2. The OR2H2 mRNA level diminished by 80% in VK2 cells treated with OR2H2 siRNA (Figure 6a). In such conditions, aldehyde 13-13 did not affect the phosphorylation levels of CAMKKβ and AMPK, nor did it alter the LC3-II to LC3-I ratio in VK2/E6E7 cells (Figure 6b–d and Figure S5a–c). This indicates that the activation of the CAMKKβ–AMPK–autophagy axis by aldehyde 13-13 is intrinsically linked to OR2H2 in VK2/E6E7 cells.

### 2.6. OR2H2 Activation Restored Senescence Induced by H_2_O_2_

Autophagy is a pivotal mechanism that counters cellular aging by upholding protein cellular homeostasis and discarding malfunctioning organelles and proteins [22]. The inhibition of cellular aging typically correlates with rejuvenated cell proliferation and curtailed cellular senescence [23]. Crucially, AMPK activation has been recognized to inhibit cellular senescence through multiple avenues. AMPK phosphorylates and restrains p27kip1, a key player in inducing apoptosis and senescence [15]. Moreover, AMPK combats senescence via the activation of SIRT1 [16]. In light of this, we examined the effect of aldehyde 13-13, which stimulates AMPK through OR2H2, on cellular senescence. 

**Figure 7:**
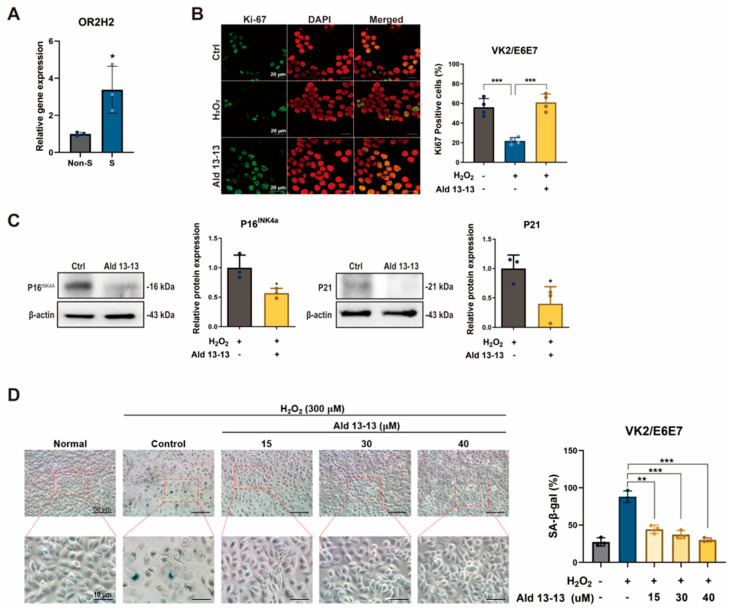
Aldehyde 13-13 acts to reduce cellular senescence: (**A**) OR2H2 expression comparison between non-senescent (Non-S) and senescent (S) cells via qPCR; (**B**) Immunocytochemical depiction of Ki-67 (green) in senescent cells exposed to aldehyde 13-13 (40 µM). DAPI is shown in red; (**C**) Immunoblot assessment of P16 and P21 expression in senescent cells treated with aldehyde 13-13; (**D**) Examination of morphology and expression of senescence-associated ß-galactosidase (SA ß-Gal) in VK2/E6E7 cells. Tests were performed in three or four replicates. Ctrl, control; Ald 13-13, aldehyde 13-13. Data are presented as mean ± SD. Two-tailed unpaired *t*-test was applied for multiple or two-group comparisons. *, *p* < 0.05; **, *p* < 0.01; ***, *p* < 0.001.

To delve deeper into OR2H2’s influence on cellular aging and senescence, we observed the repercussions of aldehyde 13-13 on senescent VK2/E6E7 cells. These cells underwent senescence induction by oxidative stress (H_2_O_2_, 300 μM) for 3 days, with a preceding incubation with aldehyde 13-13 to discern its anti-senescent capabilities. Notably, OR2H2 expression tripled in senescent cells compared to their non-senescent counterparts (Figure 7A), implying heightened sensitivity of senescent VK2/E6E7 cells to aldehyde 13-13. 

We also gauged the influence of aldehyde 13-13 on the proliferation capacity of senescent cells. A 1 h pre-treatment with aldehyde 13-13 nearly completely restored their proliferative potential, as denoted by Ki-67 immunocytochemistry, a cell proliferation marker (Figure 7B). Additionally, aldehyde 13-13 markedly lowered the expression of cellular senescence indicators, P16^INK4a^ and P21, in senescent cells (Figure 7C and Figure S6). H_2_O_2_-induced senescence amplified the expression of senescence-associated ß-galactosidase and induced changes in cell morphology (Figure 7D). However, aldehyde 13-13 administration ameliorated the senescent morphological phenotype and decreased the number of cells positive for senescence-associated ß-galactosidase (Figure 7D). Thus, OR2H2 activation by aldehyde 13-13 appears to rejuvenate cell proliferation and mitigate the H_2_O_2_-induced senescence phenotype.

### 2.7. Aldehyde 13-13 Selectively Induced Apoptosis in Senescent Cells

**Figure 8:**
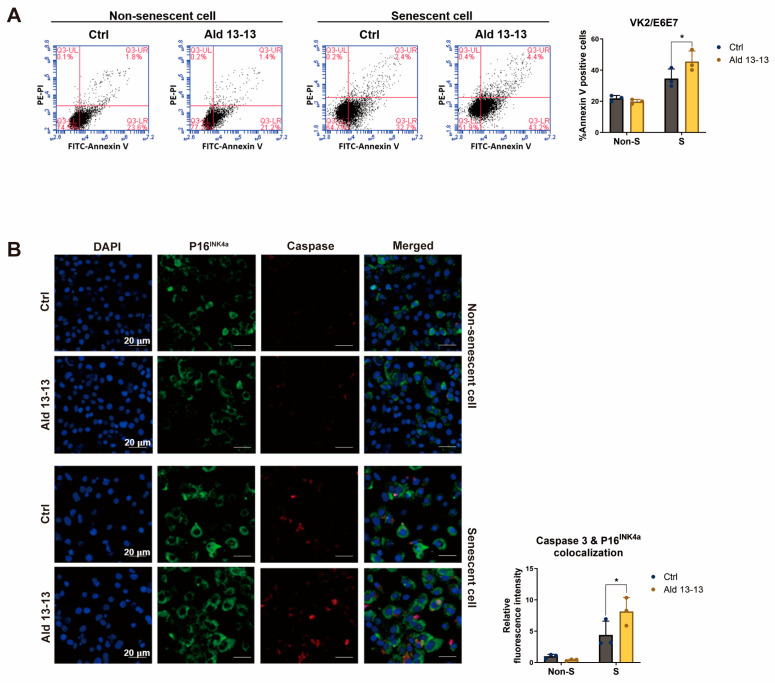
Aldehyde 13-13 showcases senolytic properties in senescent VK2 cells: (**A**) Cell sorting evaluation of Annexin V in both non-senescent (non-S) and senescent (S) cells; (**B**) Immunocytochemical study of P16^INK4a^ (green) and caspase-3 (red) in both non-senescent (non-S) and senescent (S) cells. Ctrl stands for control (0.1% DMSO); Ald 13-13 is aldehyde 13-13 (40 µM). Data are represented as mean ± SD. Two-tailed unpaired *t*-test was utilized for multiple or two-group comparisons. * *p* < 0.05.

Accumulation of senescent cells contributes to chronic diseases, tissue dysfunction, age-related ailments, and organ aging [24]. Reducing the number of these cells might enhance tissue function and lower the risk of developing age-related diseases. Some senolytic compounds, like UBX0101, selectively target senescent cells by interfering with the p53 and MDM2 interaction [25]. We subsequently examined the apoptotic effect of aldehyde 13-13 on senescent cells. Using flow cytometric analysis with Annexin V staining (Figure 8A), we found that the proportion of Annexin V positive cells remained unchanged in non-senescent cells treated with aldehyde 13-13. In contrast, the ratio increased significantly in senescent cells, suggesting aldehyde 13-13 might preferentially induce apoptosis in senescent cells compared to non-senescent ones (Figure 8A). 

To understand this effect further, we analyzed the apoptotic marker, caspase 3, in both cell types through immunocytochemistry. In non-senescent cells, both the expression of P16^INK4a^ (a senescence marker) and caspase-3 levels remained unaffected by aldehyde 13-13 (Figure 8B). Thus, the colocalization of caspase-3 and P16 appeared consistent. However, in senescent cells, while P16^INK4a^ expression markedly increased, caspase-3 levels similarly rose. Notably, aldehyde 13-13 significantly amplified the colocalization of caspase-3 and P16 in these senescent cells (Figure 8B). These results indicate that aldehyde 13-13 boosts apoptosis in senescent cells, with negligible effects in their non-senescent counterparts.

The decrease in senescent cells might enhance tissue and overall organismal function, potentially impacting lifespan. Therefore, we investigated the influence of aldehyde 13-13 on the lifespan of *C. elegans* and budding yeast. Both organisms were treated with either ethanol or 10 mM aldehyde 13-13 at 20 °C. Remarkably, aldehyde 13-13 extended the lifespan of *C. elegans* by approximately a week in comparison to controls (Figure S7a). In budding yeast, aldehyde 13-13 doses (ranging from 100 to 500 µM) notably lengthened lifespan, as evidenced by propidium iodide staining (Figure S7b) and a colony-forming unit assay (Figure S3c). These findings collectively suggest that aldehyde 13-13 enhances the lifespan of both budding yeast and *C. elegans*.

## 3. Discussion

Ectopic olfactory receptors perform varied functions across diverse organs and tissues [5,26,27]. Given that genital organs are consistently exposed to odorants, ectopic olfactory receptors might influence intracellular signaling pathways and associated physiological processes in these organs. In this study, we delved into the role of OR2H2 in VK2/E6E7 human vaginal epithelial cells.

**Figure 9:**
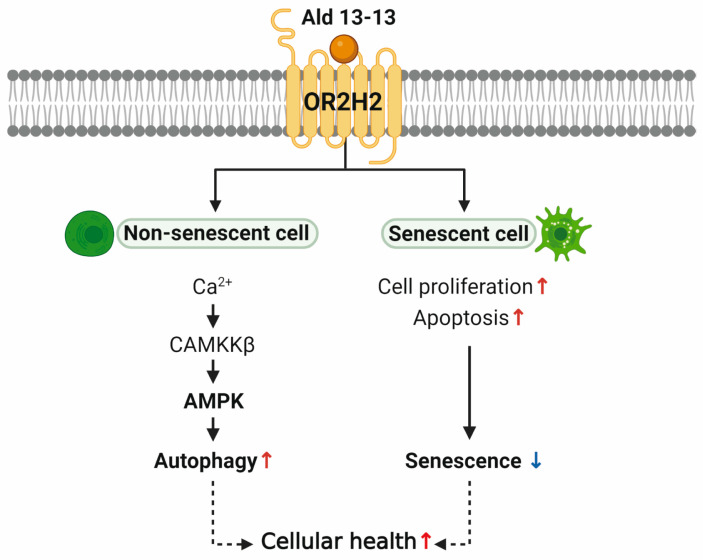
Proposed model diagram. In non-senescent cells, aldehyde 13-13 activates OR2H2, enhancing autophagy through AMPK activation. Meanwhile, in senescent cells, OR2H2 activation boosts cell proliferation and apoptosis, mitigating senescence. Collectively, this potentially promotes overall cell health.

Currently, three extant studies discuss the functionality of ectopic OR2H2, pointing to its tissue-specific roles. In human myoblasts, OR2H2 elevates the intracellular calcium concentration and diminishes myoblast fusion via Phosphoinositide 3-kinase signaling [9]. In the thyroid gland, OR2H2 expression decreases in malignant cells, compared to surrounding healthy tissues. Aldehyde 13-13 inhibits the proliferation of thyroid cancer cells [28]. The single nucleotide polymorphism of OR2H2, rs123388, is linked to celiac disease in patients from Indian and Dutch populations [29]. This polymorphism, along with five others, is associated with immune functions and Toll-like receptor signaling pathways [29]. Such findings indicate the significance of OR2H2 in gut and immune cells. Furthermore, we discerned a new function of OR2H2 in VK2/E6E7 cells. OR2H2 activation by aldehyde 13-13 energized the Ca^2+^–CAMKKβ–AMPK–autophagy signaling pathway, curbing cellular senescence via a senolytic effect (Figure 9).

Aldehyde 13-13 is a potent OR2H2 ligand [9]. Our findings also underscore its ability to robustly activate OR2H2 in VK2/E6E7 cells, thereby triggering intracellular Ca^2+^ elevations. Various effectors modulate intracellular Ca^2+^ levels, including adenylyl cyclase, which can escalate Ca^2+^ levels [30], and phospholipase C (PLC), which controls the IP_3_-DAG-Ca^2+^ signaling route [31]. Sarco/endoplasmic reticulum calcium ATPase (SERCA) channels Ca^2+^ from the cytosol to the sarcoplasmic reticulum lumen [32], and cyclic nucleotide-gated (CNG) channels facilitate Na^+^, K^+^, and Ca^2+^ ion flow [33]. Aldehyde 13-13, in our observations, amplified the intracellular Ca^2+^ concentration, an effect that was diminished by multiple calcium inhibitors like adenylyl cyclase III inhibitor (SQ22536), PLC inhibitor (U73122), SERCA inhibitor (thapsigargin), and CNG channel inhibitor (L-cis diltiazem). Thus, aldehyde 13-13’s Ca^2+^ induction in VK2/E6E7 cells likely results from the activation of several Ca^2+^ channels. A phosphokinase assay revealed the strong activation of AMPKα1 by aldehyde 13-13 in VK2/E6E7 cells, while immunoblotting illustrated the triggering of the CaMKK-β–AMPKα1–mTOR–autophagy signaling pathway due to OR2H2 activation in these cells. This pathway activation by aldehyde 13-13 was nullified in OR2H2 knockdown cells, emphasizing that OR2H2 is a functional receptor in VK2/E6E7 cells, with signaling pathway modulation by aldehyde 13-13 contingent upon OR2H2 activation.

OR2H2 activation stimulated autophagy. Metrics such as the LC3-II-to-LC3-I ratio, MDC staining, autophagic flux analysis, and transmission electron microscopy all depicted enhanced autophagy and the progression of autophagic turnover from the autophagosome to autolysosome in VK2/E6E7 cells. Autophagy stands at the crossroads of metabolic and proteostatic signaling, dictating key aging mechanisms and organism lifespan [34]. A decline in autophagic activity is believed to lead to the buildup of damaged organelles and macromolecules with aging, whereas sustaining adequate autophagic activity aids in life extension [35]. AMPK spurs autophagy either directly or indirectly through mTORC1 suppression or SIRT-1 activation. Indeed, AMPK oversees the aging process via an interconnected signaling network [36].

AMPK is a central regulator of energy metabolism and homeostasis, influencing the aging process through interactions with several signaling networks [37]. Enhanced AMPK activity potentially promotes healthspan and lifespan by mitigating cellular stress. This enhancement is achieved by activating signaling molecules such as FoxO/DAF-16, Nrf2/SKN-1, and SIRT1, stimulating the autophagic pathway via mTOR signaling, and restraining proinflammatory responses by inhibiting NF-κB signaling [36]. As AMPK activity diminishes with age, its increased activity might extend the lifespan of simpler organisms.

Aldehyde 13-13 extended the lifespan of both *C. elegans* and budding yeast, potentially through the AMPK–autophagy pathway. However, neither *C. elegans* nor budding yeast have OR2H2 homologs. As such, pinpointing the target receptor and the signaling mechanisms of aldehyde 13-13 requires further exploration. We hypothesize that in organisms like *C. elegans* and yeast, aldehyde 13-13 might counteract aging through analogous (rather than homologous) mechanisms compared to the OR2H2-dependent pathway in human cells. However, the specific anti-aging action of aldehyde 13-13 on *C. elegans* and yeast remains elusive. In humans, OR2H2, being a receptor for aldehyde 13-13, might offer therapeutic avenues for mitigating the aging of human vaginal epithelial cells. This is evidenced by the fact that Ca^2+^ and cAMP signaling via aldehyde 13-13 was specifically observed in OR2H2-expressing VK2/E6E7 cells. This potential therapeutic approach could extend to other human tissues expressing OR2H2, a possibility we aim to probe further.

AMPK maintains cellular energy balance and combats senescence [38,39]. For instance, aging skeletal muscle stem cells (MuSCs) exhibit diminished autophagy, augmented apoptosis, and reduced phosphorylation of AMPK and its downstream target, p27^Kip1^ [15]. Activating AMPK in aged MuSCs in vitro curtailed apoptosis, boosted proliferation, and enhanced transplantation efficiency in vivo. Additionally, the AMPK/p27Kip1 pathway activation lowered senescence markers in older cells, partially relying on p27^Kip1^ phosphorylation. Hence, the AMPK/p27^Kip1^ pathway might modulate the autophagy/apoptosis equilibrium in aged MuSCs, serving as a prospective target for enhancing muscle regeneration in older adults. Our results indicate that the OR2H2–AMPK–autophagy pathway activation diminishes cellular senescence in VK2/E6E7 cells. To the best of our knowledge, this constitutes the pioneering assertion that ectopic olfactory receptors can impede aging and cellular senescence.

## 4. Materials and Methods

### 4.1. Antibodies and Reagents

Primary antibodies to β-actin (SC-47778, 1:1000), CaMKK-ß (SC-50341, 1:1000), and AMPKα1/2 (SC-25792, 1:1000) were purchased from Santa Cruz Biotechnology, Inc. (Santa Cruz, CA). Primary antibodies to p-CaMKKβ (12818S, 1:500), mTOR (2983S, 1:1000), S6 ribosomal protein (2217S, 1:1000), and p-S6 ribosomal protein (2211S, 1:1000) were purchased from Cell Signaling (Danvers, MA). Anti-p-AMPKα1/2 (11183, 1:1000) and anti-p16 (41296, 1:1000) antibodies were from Signalway (College Park, MD, USA). Anti-LC3B (NB100-2220, 1:500) and anti-p21 (NBP2-29463, 1:500) antibodies were purchased from Novus (St. Louis, MO, USA). Anti-p-mTOR (ab63552) and anti-Ki-67 (ab15580) antibodies were purchased from Abcam (Cambridge, UK). Anti-mouse (31430), anti-rabbit (31460), anti-goat (31402), and Alexa Fluor 488 (A11008) immunoglobin G secondary antibodies (1:5000) were purchased from Invitrogen (Carlsbad, CA, USA). The anti-OR2H2 primary antibody (1:1000) was purchased from Antikoerper-online.de (Aachen, Germany). L-Cis diltiazem was from Abcam (Cambridge, UK). SQ22536 and thapsigargin were purchased from Enzo Life Science (Farmingdale, NY, USA) and U73122 was from Sigma-Aldrich (St. Louis, MO, USA). Aldehyde 13-13 was obtained from Henkel (Düsseldorf, Germany). A769662, an AMPK agonist, was purchased from Cayman (Ann Arbor, MI, USA). Rapamycin was obtained from LC Laboratories (Woburn, MA, USA).

### 4.2. Cell Culture

VK2/E6E7 cells (ATCC, CRL-2616) were purchased from the American Type Culture Collection (ATCC, VA, USA), and cultured in keratinocyte-serum free medium (Gibco, New York, USA, 17005042), supplemented with 0.1% human recombinant epidermal growth factor, 0.3% bovine pituitary extract, and 0.4 mM calcium chloride as instructed in the manufacturer’s protocol. Culture medium was renewed every 2–3 days. Cells were grown in 37 °C with 5% CO_2_. All compounds except L-Cis diltiazem (Abcam) and aldehyde 13-13 (Henkel) were prediluted in DMSO. L-Cis diltiazem was prediluted in distilled water and aldehyde 13-13 in ethanol. All compounds were diluted 1:1000 in growth medium or Ringer’s solution and applied to VK2/E6E7 cells. All dilutions of aldehyde 13-13 were prepared fresh before use.

### 4.3. RNA Extraction and RT-PCR

Total cellular RNA was extracted with Total RNA extraction reagent RNAiso Plus (1 mL, Takara Bio, 9109, Japan) according to the manufacturer’s instructions. Complementary DNA was synthesized from 1 μg RNA with ReverTra Ace^®^ RT Master Mix kit (Toyobo, Osaka, Japan, FSQ-301) according to the manufacturer’s instructions. One μg of cDNA was then used as a template for PCR reactions and specific primers were designed by the Primer-BLAST designer program (NCBI, Bethesda, MD, USA). RT-PCR was performed with EmeraldAmp^®^ GT PCR Master Mix reagent according to the manufacturer’s instructions. The RT-PCR products were analyzed by agarose gel electrophoresis (3%) and image analysis was obtained by using a ChemiDoc touch imaging system with the Image Lab 5.2 software (Bio-Rad, Redmond, WA, USA). L32 was used to normalize the data.

### 4.4. Real Time qPCR

After a 24 h treatment in 6-well plates, total cellular RNA was extracted using the RNAiso Plus reagent (Takara Bio, Shiga, Japan), following the manufacturer’s guidelines. Complementary DNA (cDNA) was synthesized from 1 μg of RNA using the ReverTra Ace^®^ RT Master Mix kit (FSQ-301; Toyobo, Japan), again adhering to the manufacturer’s protocol. An amount of 1 μg of the resultant cDNA was utilized as a template for the polymerase chain reaction (PCR). Specific primers were crafted using the Primer-BLAST designer tool (NCBI, Bethesda, MD, USA). The cDNA was then subjected to PCR using the iQ5 i-Cycler system (Bio-Rad Laboratories, Hercules, CA, USA) and the Thunderbird SYBR^®^ qPCR Mix reagent (T00PS-201; Takara Bio). Amplification parameters for the cDNA included an initial 30 s denaturation at 95 °C, 40 cycles of 10 s denaturation at 95 °C, 20 s annealing at 59 °C, and 20 s extension at 68 °C. The fluorescent signal was quantified automatically post each PCR cycle. Gene expression was determined from the threshold cycle (Ct value), and L32 was employed for normalization. The oligonucleotide sequences can be found in Supplementary Table S1.

### 4.5. OR2H2 Immunocytochemical Staining

Immunocytochemical staining was carried out using immunofluorescence microscopy. VK2/E6E7 cells were placed on coverslips inside a 24-well plate, containing 500 μL of cell culture medium, and incubated at 37 °C in a 5% CO_2_ environment. Upon reaching 80% confluence, the medium was discarded, and the cells were rinsed with PBS. Post-fixation in ice-cold acetone, cells were treated with both primary (1:100, anti-OR2H2; Antikorperonline.de) and secondary antibodies (1:1000, goat anti-rabbit 546; Thermo Fisher, Carlsbad, CA, USA). Subsequently, cells were exposed to Alexa Fluor phalloidin 488 (1:200, Thermo Fisher) for 45 min at ambient temperature with gentle agitation. The cells were then sealed with Immu-Mount solution (Thermo Fisher) and inspected under an LSM 510 Meta Confocal Microscope (Carl Zeiss, Jena, Germany) employing a 40× oil immersion objective and the Leica Application Suite X Software. The specificity of the antibodies utilized was previously ascertained by rho-tagged transfection of Hana3A cells [40].

### 4.6. Calcium Imaging and Calcium Inhibitor Treatments

Intracellular calcium concentration changes were monitored using the fluorescent dye Fura-2-acetoxylmethyl ester (Fura-2/AM; Invitrogen). VK2/E6E7 cells were cultured in 35 mm dishes and incubated in growth medium containing 3 μM Fura-2/AM for 30 min at 37 °C, protected from light. Next, growth medium was replaced with Ringer’s solution. Aldehyde 13-13 and calcium inhibitors were dissolved in Ringer’s solution (1:1000) and applied directly to the cells. In desensitization experiments, the cells were stimulated repetitively with aldehyde 13-13 (300 µM) for 2 min while control cells were treated with vehicle (ethanol in Ringer’s solution) repeatedly for 2 min. In experiments under calcium-free conditions, cells were stimulated with aldehyde 13-13 (500 µM) for 3 min, then washed for 3 min with Ringer’s solution. Cells were then treated with calcium-free Ringer’s solution for 7 min, and then aldehyde 13-13 was added for 3 min. A third application of aldehyde 13-13 was performed for 3 min. In experiments with calcium channel inhibitors, cells were stimulated with aldehyde 13-13 (500 µM) for 3 min. After 2 min washing with Ringer’s solution, the cells were stimulated with the calcium channel inhibitors. Subsequently, aldehyde 13-13 and inhibitors were co-applied for 3 min. A third application was also prepared under normal conditions after a 2 min wash-out step. Calcium channel inhibitors were incubated under the following conditions: SQ 22536 (10 µM) for 10 min; L-cis diltiazem (100 µM) for 3 min; U 73122 (5 µM) for 5 min; thapsigargin (5 µM) for 25 min. Intracellular calcium flux was observed using a DMI, 6000CS inverse microscope (Leica, Wetzlar, Germany), and calcium-dependent fluorescence changes were detected using a 10-fold fluorescence objective. Fluorescence emission intensities (340 and 380 nm) were detected by a DFX 360FX CCD camera (Leica). Regions of interest (ROIs) were analyzed using Advanced Fluorescence software LASX Office 1.4.5 27713 (LAS AF; Leica).

### 4.7. Phosphokinase Array

The human phosphokinase array kit by RnD systems was applied to investigate the phosphorylation of 43 different kinases under the influence of aldehyde 13-13 stimulation for an incubation time of 30 min. Before starting the array, according to the manufacturer’s instructions, protocol cells were cultivated in 75 cm^2^ culture flasks until a confluence of 90% was achieved. Medium was exchanged with fresh medium containing the diluted aldehyde 13-13 in a concentration of 500 µM. The control was treated with ethanol (<0.1%). After incubation, medium was aspirated, cells were covered with PBS and manually detached with a cell scraper. The detached cells were then subsequently taken up in the PBS and centrifuged for 10 min at 1000 rpm and 4 °C. The cell pellets thus obtained were then lysed in RIPA buffer and their protein concentration was determined using the Pierce^TM^ BCA protein assay kit. The total amount of protein was 250 µg for the treated and untreated samples. The evaluation of the membrane was performed by the chemiluminescence System Fusion SL 3500-WL. The intensity of the individual array dots was analyzed with the program Image J and a microarray plugin and normalized to the control.

### 4.8. OR2H2 Knockdown

For mRNA interference of *OR2H2*, VK2 cells (4 × 10^5^) were seeded in six-well plates and transfected with a siRNA against *OR2H2* (Cat No. sc-95450; Santa Cruz Biotechnology, Inc.) using Lipofectamine 2000 according to the manufacturer’s instructions. Briefly, cells were cultured with siRNA and Lipofectamine 2000 in Opti-MEM reduced-serum medium (Cat No. 32985070; Gibco, New York, NY, USA). After incubation for 5 h at 37 °C in a CO_2_ atmosphere, medium was removed, and cells were incubated for 19 h in serum-containing medium at 37 °C. To verify transfection, VK2 cells transfected with fluorescein-conjugated control siRNA (Cat No. SN1021; Bioneer, Dae-Jeon, Republic of Korea) were harvested. Intracellular fluorescence intensity was measured using a flow cytometer equipped with an FL1 detector (BD Biosciences, Franklin Lakes, NJ, USA).

### 4.9. cAMP Assay

Experiments were performed as previously reported (Wu et al., 2021). To measure intracellular cyclic adenosine monophosphate (cAMP) concentration changes, cells were cultivated in a 96-well plate until 90% confluence. Aldehyde 13-13 was diluted in induction buffer, consisting of Ringer’s solution, 500 μM isobutyl-1-methylxanthin, and 100 μM 4-imidazolidin-2-one (Sigma-Aldrich). After incubation for 30 min, intracellular cAMP concentrations were measured using cAMP Glo Assay Kit (Promega) and Fusion α Multi-well Plate Reader (Packard Bioscience, Wellesley, MA, USA). Data were normalized to the solvent control (ethanol). Treatment for 30 min with 10 μM forskolin (Sigma-Aldrich) served as the positive control.

### 4.10. Cell Viability Assay

The viability of siRNA-transfected VK2/E6E7 cells was assessed using a 2,5-diphenyl-2H-tetrazolium bromide (MTT) assay [41]. Cells were seeded at a density of 1 × 10^4^ cells per well in 96-well culture plates and were allowed to adhere overnight. The following day, the culture medium was discarded and replaced with a medium containing 10% MTT (Sigma Aldrich). Cells were then incubated for 3 h at 37 °C. Afterward, the MTT solution was carefully removed from each well, and dimethyl sulfoxide (DMSO; Bio Basic Inc., Markham, ON, Canada) was added to dissolve the formazan crystals. The absorbance of each sample was subsequently measured at 570 nm using a microplate spectrophotometer (Multiskan Go; Thermo Fisher Scientific).

### 4.11. Immunoblot Analysis

Total cellular proteins were extracted using radioimmunoprecipitation assay buffer supplemented with Halt protease and phosphatase inhibitor reagent (Thermo Fisher Scientific, Carlsbad, CA, USA). Total cellular proteins were centrifuged for 20 min at 12,000 rpm to obtain the soluble protein fraction. Protein concentration was measured using a protein assay dye reagent concentrate (Bio-Rad). Denatured proteins were run on a sodium dodecyl sulfate-polyacrylamide gel, and transferred to nitrocellulose membranes (GVS Filter Technology, Morecambe, UK). The membranes were blocked in TBS with 5% (*w*/*v*) non-fat dried milk for 1 h at room temperature. The primary antibodies were diluted in TBS-Tween 20 (0.1%) with 5% (*w*/*v*) non-fat dried milk. The secondary antibodies were diluted in TBS with 0.1% Tween-20. Immunoblot images were obtained using the ChemiDoc Touch Imaging System and analyzed with Image Lab 5.2 software (Bio-Rad). Protein levels were normalized with β-actin after stripping the identical blot using stripping buffer (Dyne Bio, CBS3180, Seong-Nam, Republic of Korea).

### 4.12. mRFP-GFP-LC3 Puncta Formation Assay

VK2/E6E7 cells (2 × 10^5^) were seeded in a six-well confocal plate. After 24 h, cells were transfected with 1.5 ng of mRFP-GFP-LC3 plasmid using DharmaFECT transfection reagent (Horizon Discovery, Cambridge, UK) as per the manufacturer’s instructions and incubated for 48 h. Next, cells were treated with ethanol (control) or 500 µM aldehyde 13-13 for 30 min. Cells were washed once with PBS and fixed with 4% paraformaldehyde for 10 min. mRFP and GFP fluorescent puncta were observed under an LSM510 META confocal microscope and images were analyzed with LSM700 version 3.2 software (Carl Zeiss, Jena, Germany).

### 4.13. Monodansylcadaverine Staining Assay

VK2/E6E7 cells were seeded in a black or clear 96-well plate at 1 × 10^4^ per well. After 24 h, cells were treated with ethanol, rapamycin (100 nM), or aldehyde 13-13 (500 µM) for 30 min and autophagy vacuoles were stained using a monodansylcadaverine (MDC) staining kit (Cayman, Ann Arbor, MI) as per the manufacturer’s protocol. Autophagy vacuoles were detected under a fluorescence microscope (Nikon Eclipse Ti) and images were analyzed using NIS-elements F software (Nikon Instruments, Inc., New York, NY, USA).

### 4.14. Autophagy Analysis by Transmission Electron Microscopy

VK2 cells (5 × 10^6^) were cultured in 175T flasks for 24 h and treated with aldehyde 13-13 (500 µM) for 30 min. For TEM, cells were fixed with Karnovsky’s fixative and stored at 4 °C overnight. Cells were post-fixed with 1% osmium tetroxide and stained with 0.5% uranyl acetate overnight followed by dehydration in an ethanol gradient. The cells were infiltrated with Spurr’s resin overnight and visualized using a JEM1010 electron microscope at 120 kV (JEOL, Tokyo, Japan).

### 4.15. Senescence-Associated β-Galactosidase Staining

VK2/E6E7 cells were seeded in 24-well plates at 5 × 10^4^/well. Cells were grown for 24 h and treated with ethanol (control) or aldehyde 13-13 (15, 30, or 40 µM) for 1 h before H_2_O_2_ (300 µM) treatment. Next, the cells were incubated at 37 °C in 5% CO_2_ for 3 days to induce senescence.

### 4.16. Ki-67 Immunocytochemical Staining

VK2/E6E7 cells (3 × 10^4^) were seeded on glass coverslips for 24 h. Cells were washed twice with PBS and fixed with 4% paraformaldehyde for 10 min. Fixed cells were incubated with 5% bovine serum albumin (BSA) in TBS-Tween 20 (0.1%) for 45 min and then with Ki-67 antibody (1:500, Abcam, UK) overnight at 4 °C. Next, cells were incubated with the Alexa Fluor 488-tagged anti-IgG secondary antibody (1:1000; Invitrogen) for 1 h in the dark. After washing, nuclei were stained with DAPI (Invitrogen) for 5 min. Coverslips were embedded in anti-fade mounting solution (Thermo Fisher Scientific) and slides were visualized using an LSM510 Meta confocal microscope and LSM700 v. 3.2 software (Carl Zeiss).

### 4.17. Annexin V and Propidium Iodide Staining

Cells were seeded in a six-well plate at 3 × 10^5^/well and grown for 24 h. Proliferating cells were treated with vehicle and 40 µM aldehyde 13-13. Senescent cells were cotreated with 200 µM H_2_O_2_ plus vehicle and aldehyde 13-13. Next, cells were stained and incubated with 3 µL Annexin-V-FITC (BD Biosciences, CA, USA) and 10 µL propidium iodide (BD Biosciences, CA, USA) for 20 min and apoptosis analysis was performed using Accuri C6 Plus FACS (BD Biosciences).

### 4.18. Budding Yeast and Culture Conditions

*Saccharomyces cerevisiae* BY4741 (MATa his3Δ1 leu2Δ0 met15Δ0, ura3Δ0) was streaked onto yeast extract peptone-dextrose (YPD) agar containing 2% Bacto agar, 1% Bacto yeast extract, 2% Bacto peptone, and 2% Difco dextrose (BD Biosciences). The plates were incubated at 30 °C to obtain isolated single colonies. A few picked colonies were inoculated into 10 mL of YPD medium (1% Bacto yeast extract, 2% Bacto peptone, and 2% Difco dextrose), and cultured overnight to make a seed culture. The seed culture was inoculated into 20 mL of YPD medium to an optical density at 600 nm of 0.2. All yeast cultures were incubated at 30 °C in an orbital shaker at 200 rpm.

### 4.19. Measurement of Chronological Lifespan by PI Staining and Flow Cytometry

Yeast cells were harvested by centrifugation, washed by resuspending in 1 mL of phosphate-buffered saline (PBS) and incubated for 20 min at 30 ℃ after adding 5 µL of PI solution (1 mg/mL, Sigma Aldrich). Stained cells were analyzed using a flow cytometer (FACS Verse; Becton Dickinson) with excitation at 488 nm and emission at 585 nm. Approximately 20,000 cells were analyzed per sample, and data were analyzed using Cell Quest software v3.3 (Becton Dickinson).

### 4.20. Colony-Forming Unit Assay of Budding Yeast

A colony-forming unit (CFU) assay was performed to determine yeast viability. Yeasts were harvested, serially diluted in PBS, counted using a hemocytometer, and 200 cells were spread onto YPD agar and incubated at 30 °C until colony formation.

### 4.21. Caenorhabditis elegans (C. elegans) Longevity Assay

*C. elegans* strains were cultured on fresh NGM plates for 2–3 generations without starvation, and lifespan analysis was conducted at 20 °C. Worms were synchronized by isolating eggs from gravid adults using hypochlorite and NaOH. When worms reached the L4 stage, 2′-deoxy-5-fluorouridine (FUdR, 0.6 mM) was added to prevent internal hatching. Aldehyde 13-13 (10 mM) was added to each well along with OP50 on the first day of the lifespan assay. Ethanol (0.1%) was vehicle in the control group. Wells containing > 15 worms were excluded from the analysis because of the risk of dietary restriction. Worm movement was used to determine whether the animals were alive or dead. Survival curves were analyzed by the log-rank (Mantel–Cox) method.

### 4.22. Statistical Analysis

Results are means ± standard error of the mean (SEM). To generate dose–response curves, SigmaPlot 10.0 was used, and curves were fitted using the Hill three-parameter equation. Significance was examined by two-tailed unpaired *t*-test. Data were subjected to one-way ANOVA followed by Tukey’s HSD test or Student’s *t*-test for multiple or two-group comparisons. Different letters indicate a significant difference at *p* < 0.05 (ANOVA).

## 5. Conclusions

In non-senescent cells, aldehyde 13-13 heightened autophagy by AMPK activation and, in senescent cells, increased cell proliferation while inducing apoptosis, thus reducing senescence (Figure 9). Our data from VK2/E6E7 cells suggest that aldehyde 13-13 might enhance cellular health and counteract aging in the human vaginal epithelium through AMPK activation. AMPK appears to hold pivotal roles in cellular senescence and apoptosis. For instance, in mouse embryonic fibroblasts, removing AMPKα2 accelerates cellular senescence and cell cycle arrest triggered by oxidative stress-induced senescence [42]. Activation of AMPK by specific molecules can drive cancer cell apoptosis. This process stimulates apoptosis by inhibiting AKT and COX-2, repressing mTORC1, and modulating P53 and P21. Our cumulative data show that aldehyde 13-13 activates OR2H2 in VK2/E6E7 cells, induces the AMPK–autophagy pathway, curtails cellular senescence while promoting apoptosis, and potentially extends cell lifespan (Figure 9). Human OR2H2 might represent a novel target in the fight against aging and cellular senescence (Figure 9).

## Data Availability

The data that support the findings of this study are available from the corresponding author upon reasonable request.

## Supplementary Materials

The following supporting information can be downloaded at https://www.mdpi.com/article/10.3390/ph16091221/s1, Figure S1: Intracellular Ca^2+^ response upon propionic acid and lactic acid stimulation; Figure S2: Results of phosphokinase array; Figure S3: Full length uncropped western blot images used to generate Figure 4; Figure S4: Full length uncropped westernblot images used to generate Figure 5A; Figure S5: Full length uncropped westernblot images used to generate Figure 6b–d; Figure S6: Full length uncropped westernblot images used to generate Figure 7B; Figure S7: Effect of aldehyde 13-13 on the lifespan of *C. elegans* and budding yeast; Table S1: qPCR primers.

## References

[1] Feldmesser E., Olender T., Khen M., Yanai I., Ophir R., Lancet D. (2006). Widespread ectopic expression of olfactory receptor genes. BMC Genom..

[2] Flegel C., Manteniotis S., Osthold S., Hatt H., Gisselmann G. (2013). Expression profile of ectopic olfactory receptors determined by deep sequencing. PLoS ONE.

[3] Busse D., Kudella P., Gruning N.M., Gisselmann G., Stander S., Luger T., Jacobsen F., Steinstrasser L., Paus R., Gkogkolou P. (2014). A synthetic sandalwood odorant induces wound-healing processes in human keratinocytes via the olfactory receptor OR2AT4. J. Investig. Dermatol..

[4] Cheret J., Bertolini M., Ponce L., Lehmann J., Tsai T., Alam M., Hatt H., Paus R. (2018). Olfactory receptor OR2AT4 regulates human hair growth. Nat. Commun..

[5] Lee S.J., Depoortere I., Hatt H. (2019). Therapeutic potential of ectopic olfactory and taste receptors. Nat. Rev. Drug Discov..

[6] Spehr M., Gisselmann G., Poplawski A., Riffell J.A., Wetzel C.H., Zimmer R.K., Hatt H. (2003). Identification of a testicular odorant receptor mediating human sperm chemotaxis. Science.

[7] Shirokova E., Schmiedeberg K., Bedner P., Niessen H., Willecke K., Raguse J.D., Meyerhof W., Krautwurst D. (2005). Identification of specific ligands for orphan olfactory receptors. G protein-dependent agonism and antagonism of odorants. J. Biol. Chem..

[8] Chiarelli F., Di Marzio D. (2008). Peroxisome proliferator-activated receptor-gamma agonists and diabetes: Current evidence and future perspectives. Vasc. Health Risk Manag..

[9] Kalbe B., Osterloh M., Schulz V.M., Altmuller J., Becker C., Osterloh S., Hatt H. (2018). OR2H2 regulates the differentiation of human myoblast cells by its ligand aldehyde 13-13. Arch. Biochem. Biophys..

[10] Parish S.J., Nappi R., Krychman M.L., Spadt K., Simon J., Goldstein J., Kingsberg S. (2013). Impact of vulvovaginal health on postmenopausal women: A review of surveys on symptoms of vulvovaginal atrophy. Int. J. Women’s Health.

[11] Shao Q., Liu T., Wang W., Duan Q., Liu T., Xu L., Huang G., Chen Z. (2020). The Chinese herbal prescription JZ-1 induces autophagy to protect against herpes simplex Virus-2 in human vaginal epithelial cells by inhibiting the PI3K/Akt/mTOR pathway. J. Ethnopharmacol..

[12] Yang S., Traore Y., Jimenez C., Ho E.A. (2019). Autophagy induction and PDGFR-beta knockdown by siRNA-encapsulated nanoparticles reduce chlamydia trachomatis infection. Sci. Rep..

[13] Leiblum S., Bachmann G., Kemmann E., Colburn D., Swartzman L. (1983). Vaginal atrophy in the postmenopausal woman. The importance of sexual activity and hormones. JAMA.

[14] Alers S., Loffler A.S., Wesselborg S., Stork B. (2012). Role of AMPK-mTOR-Ulk1/2 in the regulation of autophagy: Cross talk, shortcuts, and feedbacks. Mol. Cell Biol..

[15] White J.P., Billin A.N., Campbell M.E., Russell A.J., Huffman K.M., Kraus W.E. (2018). The AMPK/p27(Kip1) Axis Regulates Autophagy/Apoptosis Decisions in Aged Skeletal Muscle Stem Cells. Stem Cell Rep..

[16] Xu W., Luo Y., Yin J., Huang M., Luo F. (2023). Targeting AMPK signaling by polyphenols: A novel strategy for tackling aging. Food Funct..

[17] Finkel T., Deng C.X., Mostoslavsky R. (2009). Recent progress in the biology and physiology of sirtuins. Nature.

[18] Mihaylova M.M., Shaw R.J. (2011). The AMPK signalling pathway coordinates cell growth, autophagy and metabolism. Nat. Cell Biol..

[19] Rocchi A., He C. (2015). Emerging roles of autophagy in metabolism and metabolic disorders. Front. Biol..

[20] Hansen M., Rubinsztein D.C., Walker D.W. (2018). Autophagy as a promoter of longevity: Insights from model organisms. Nat. Rev. Mol. Cell Biol..

[21] Blommaart E.F.C., Luiken J.J.F.P., Blommaart P.J.E., Vanwoerkom G.M., Meijer A.J. (1995). Phosphorylation of Ribosomal-Protein S6 Is Inhibitory for Autophagy in Isolated Rat Hepatocytes. J. Biol. Chem..

[22] Lopez-Otin C., Blasco M.A., Partridge L., Serrano M., Kroemer G. (2013). The hallmarks of aging. Cell.

[23] Ogrodnik M. (2021). Cellular aging beyond cellular senescence: Markers of senescence prior to cell cycle arrest in vitro and in vivo. Aging Cell.

[24] Song P., An J.Q., Zou M.H. (2020). Immune Clearance of Senescent Cells to Combat Ageing and Chronic Diseases. Cells.

[25] Jeon O.H., Kim C., Laberge R.M., Demaria M., Rathod S., Vasserot A.P., Chung J.W., Kim D.H., Poon Y., David N. (2017). Local clearance of senescent cells attenuates the development of post-traumatic osteoarthritis and creates a pro-regenerative environment. Nat. Med..

[26] Massberg D., Hatt H. (2018). Human Olfactory Receptors: Novel Cellular Functions Outside of the Nose. Physiol. Rev..

[27] Tong T., Wang Y., Kang S.G., Huang K. (2021). Ectopic Odorant Receptor Responding to Flavor Compounds: Versatile Roles in Health and Disease. Pharmaceutics.

[28] Weidinger D., Jovancevic N., Zwanziger D., Theurer S., Hones J., Fuhrer D., Hatt H. (2021). Functional Characterization of Olfactory Receptors in the Thyroid Gland. Front. Physiol..

[29] Banerjee P., Bhagavatula S., Sood A., Midha V., Thelma B.K., Senapati S. (2019). Association study identified biologically relevant receptor genes with synergistic functions in celiac disease. Sci. Rep..

[30] Tang W.J., Hurley J.H. (1998). Catalytic mechanism and regulation of mammalian adenylyl cyclases. Mol. Pharmacol..

[31] Putney J.W., Tomita T. (2012). Phospholipase C signaling and calcium influx. Adv. Biol. Regul..

[32] Primeau J.O., Armanious G.P., Fisher M.E., Young H.S. (2018). The SarcoEndoplasmic Reticulum Calcium ATPase. Subcell. Biochem..

[33] Dzeja C., Hagen V., Kaupp U.B., Frings S. (1999). Ca^2+^ permeation in cyclic nucleotide-gated channels. EMBO J..

[34] Klionsky D.J., Abdel-Aziz A.K., Abdelfatah S., Abdellatif M., Abdoli A., Abel S., Abeliovich H., Abildgaard M.H., Abudu Y.P., Acevedo-Arozena A. (2021). Guidelines for the use and interpretation of assays for monitoring autophagy (4th edition). Autophagy.

[35] Barbosa M.C., Grosso R.A., Fader C.M. (2018). Hallmarks of Aging: An Autophagic Perspective. Front. Endocrinol..

[36] Saikia R., Joseph J. (2021). AMPK: A key regulator of energy stress and calcium-induced autophagy. J. Mol. Med..

[37] Salminen A., Kaarniranta K. (2012). AMP-activated protein kinase (AMPK) controls the aging process via an integrated signaling network. Ageing Res. Rev..

[38] Yang S., Long L.H., Li D., Zhang J.K., Jin S., Wang F., Chen J.G. (2015). beta-Guanidinopropionic acid extends the lifespan of Drosophila melanogaster via an AMP-activated protein kinase-dependent increase in autophagy. Aging Cell.

[39] Zhao X., Zeng Z., Gaur U., Fang J., Peng T., Li S., Zheng W. (2019). Metformin protects PC12 cells and hippocampal neurons from H_2_O_2_ -induced oxidative damage through activation of AMPK pathway. J. Cell Physiol..

[40] Flegel C., Schöbel N., Altmüller J., Becker C., Tannapfel A., Hatt H., Gisselmann G. (2015). RNA-Seq Analysis of Human Trigeminal and Dorsal Root Ganglia with a Focus on Chemoreceptors. PLoS ONE.

[41] Solis-Sanchez D., Rivera-Piza A., Lee S., Kim J., Kim B., Choi J.B., Kim Y.W., Ko G.P., Song M.J., Lee S.-J. (2020). Antiviral Effects of *Lindera obtusiloba* Leaf Extract on Murine Norovirus-1 (MNV-1), a Human Norovirus Surrogate, and Potential Application to Model Foods. Antibiotics.

[42] Ding Y., Chen J., Okon I.S., Zou M.H., Song P. (2016). Absence of AMPKα2 accelerates cellular senescence via p16 induction in mouse embryonic fibroblasts. Int. J. Biochem. Cell Biol..

