# Supplementary material for: OR2H2 Activates CAMKKβ–AMPK–Autophagy Signaling Axis and Suppresses Senescence in VK2/E6E7 Cells
Source: Pharmaceuticals (Basel). 2023 Aug 29;16(9):1221. doi: 10.3390/ph16091221 (PMC10535153; doi:10.3390/ph16091221)
Supplement: Supplementary file 1 [file pharmaceuticals-16-01221-s001.zip › pharmaceuticals-2527875-supplementary.pdf]

Supplementary Information to:

OR2H2 Activates CAMKKβ–AMPK–Autophagy Signaling Axis and Suppresses Senescence in VK2/E6E7 Cells

Ji Min Kim [1,†], Sina Dziobaka [2,†], Ye Eun Yoon [1,†], Ha Lim Lee [1], Ji Hyun Jeong [1], In-Ryeong Lee [1], Daniel Weidinger [2], Changwon Yang [1,3], Deokho Kim [1,3], Yalcin Gulperi [1,3], Cheol-Koo Lee [1,3], Jeongwon Sohn [4,5], Gwonhwa Song [1,3], Hanns Hatt [2,*] and Sung-Joon Lee [6,7,8,*]

1 Department of Biotechnology, School of Life Science and Biotechnology for BK21 PLUS, Korea University, Seoul 02855, Republic of Korea; jamiekim19@gmail.com (J.M.K.); yeeun916@korea.ac.kr (Y.E.Y.); hagurim94@naver.com (H.L.L.); embroider13@naver.com (J.H.J.); lee3231817@naver.com (I.-R.L.); ycw117@korea.ac.kr (C.Y.); mzxcmz@naver.com (D.K.); gulperiyalcin@korea.ac.kr (Y.G.); cklee2005@korea.ac.kr (C.-K.L.)
2 Department of Cell Physiology, Ruhr-University Bochum, 44791 Bochum, Germany; sina.dziobaka@rub.de (S.D.); daniel.weidinger@rub.de (D.W.)
3 Division of Biotechnology, College of Life Sciences and Biotechnology, Korea University, Seoul 02855, Republic of Korea
4 Department of Biochemistry and Molecular Biology, Korea University College of Medicine, Seoul 02842, Republic of Korea; biojs@korea.ac.kr
5 Korea Institute of Molecular Medicine and Nutrition, Seoul 02842, Republic of Korea
6 Department of Food Bioscience and Technology, College of Life Sciences and Biotechnology, Korea University, Seoul 02855, Republic of Korea
7 Interdisciplinary Program in Precision Public Health, Korea University, Seoul 02846, Republic of Korea
8 BK21 Four Institute of Precision Public Health, Korea University, Seoul 02846, Republic of Korea
* Correspondence: hanns.hatt@rub.de (H.H.); junelee@korea.ac.kr (S.-J.L.)
† These authors contributed equally to this work.

Table S1. qPCR primers

Gene	Forward 5’-3’	Reverse 3’-5’	Product size (bp)
OR2H2	GGTCCCAGCTCTAATTCGACT	CACTGCCCAGGTAATGGCTC	136
OR2H1	TGCAAGAACTGAACCGACCA	ACCTCACATTGGTGCCTCTG	194
OR51E1	GATTGTCCATGGTGCATCGC	TGCGCTGTCGAATCTCCTTT	138
OR51E2	CATATTTGCAAGCTCGGCCC	GGCATGTGTGAAGTTGCAGG	172
OR1D2	AGAGCAGTGGGACTTTTGGT	GCCAGGAAGAAGTACACGGG	251
OR2J3	GCATGTATCTCCAGCCACCA	CATACACAGGCTCATTCCCAC	169
OR2B6	CAGATCGACCTTGGCTGGAG	GGGCTACACAGCCACGATAA	246
OR2A4/7	TCCTACAGCCCCCTTCTAGG	GGAATGCCGATCCATGATGA	215

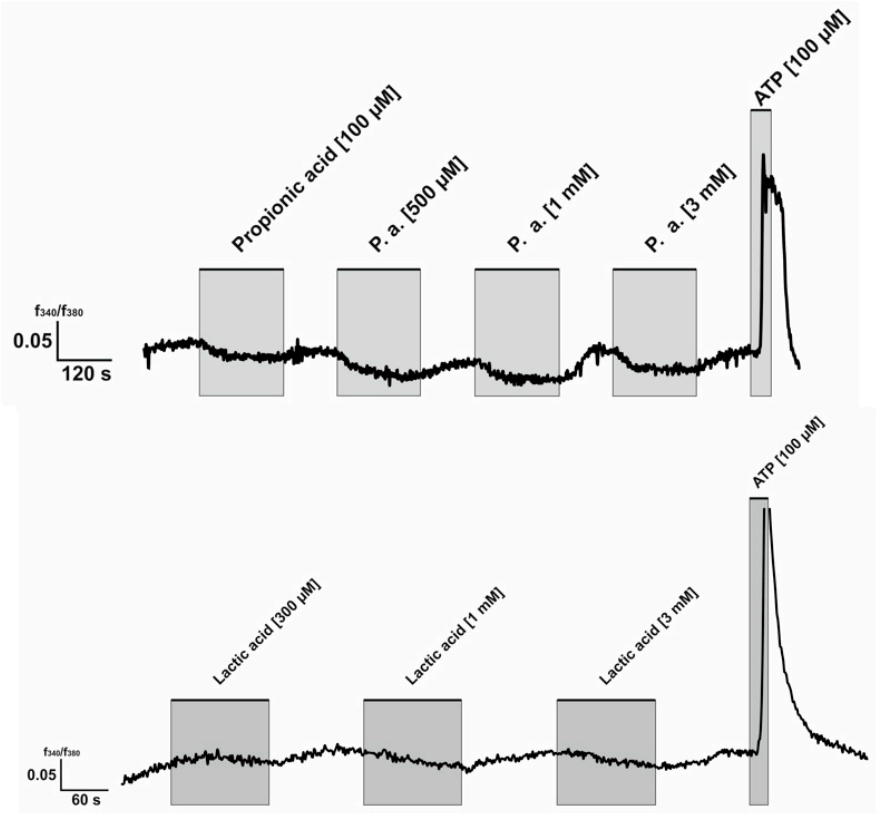

Figure S1. Intracellular Ca^{2+} response upon propionic acid (100, 500 µM and 1, 3 mM) and lactic acid (300 µM and 1, 3 mM) stimulation. Application time was labeled by the grey square. ATP (100 µM) was applied as a positive control.

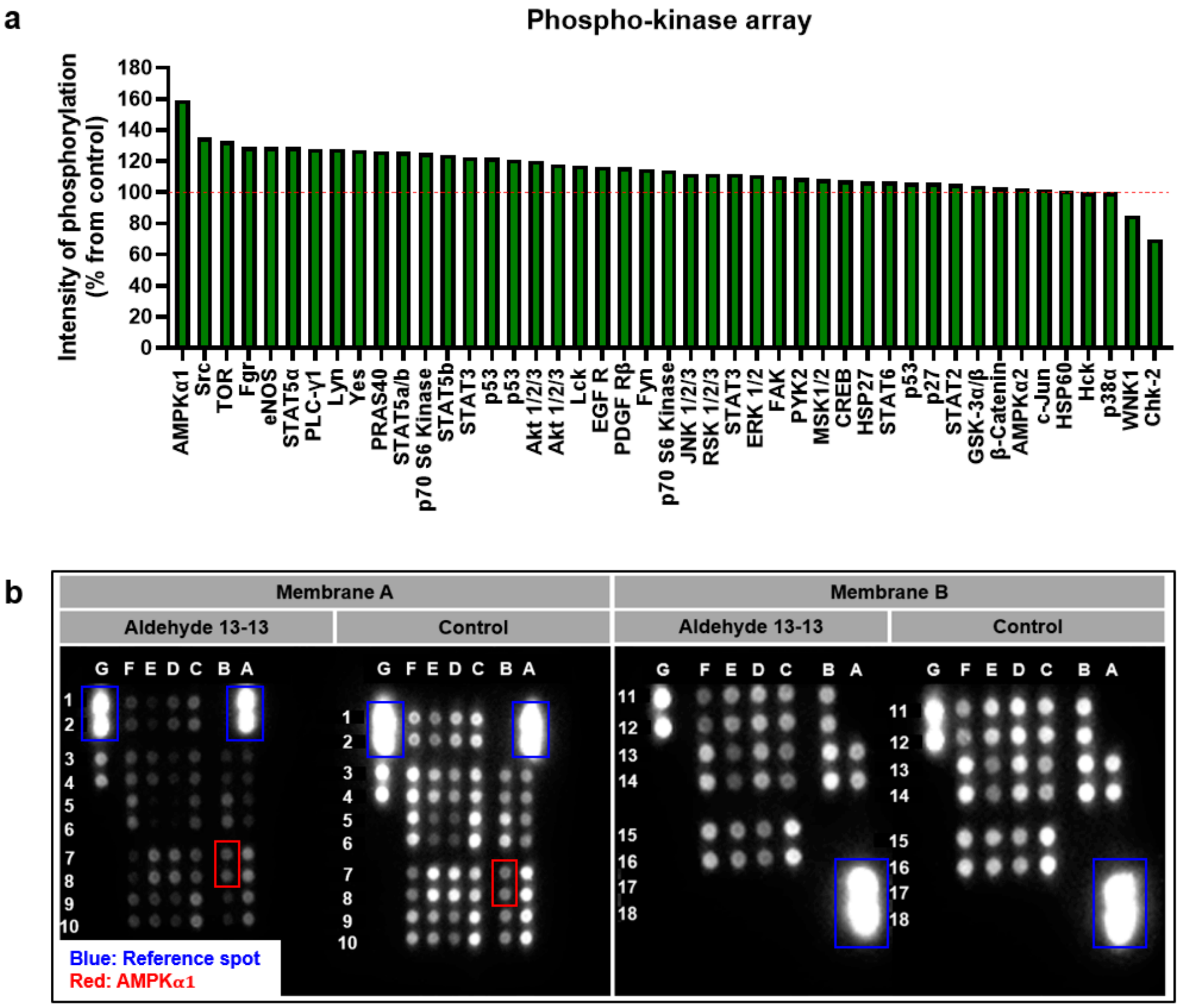

Membrane A			
**Row**	**Column**	**Target**	**Phosphorylation site**
1	A	Reference spot	-
1	B	-	-
1	C	TOR	S2448
1	D	Src	Y419
1	E	Fyn	Y420
1	F	Hck	Y411
1	G	Reference spot	-
2	A	Reference spot	-
2	B	-	-
2	C	TOR	S2448
2	D	Src	Y419
2	E	Fyn	Y420
2	F	Hck	Y411
2	G	Reference spot	-
3	A	p38α	T180/Y182
3	B	EGF R	Y1086
3	C	CREB	S133

3	D	Lyn	Y397
3	E	Yes	Y426
3	F	Chk-2	T68
3	G	PRAS40	T246
4	A	p38α	T180/Y182
4	B	EGF R	Y1086
4	C	CREB	S133
4	D	Lyn	Y397
4	E	Yes	Y426
4	F	Chk-2	T68
4	G	PRAS40	T246
5	A	ERK1/2	T202/Y204, T185/Y187
5	B	MSK1/2	S376/S360
5	C	HSP27	S78/S82
5	D	Lck	Y394
5	E	Fgr	Y412
5	F	FAK	Y397
5	G	-	-
6	A	ERK1/2	T202/Y204, T185/Y187
6	B	MSK1/2	S376/S360
6	C	HSP27	S78/S82
6	D	Lck	Y394
6	E	Fgr	Y412
6	F	FAK	Y397
6	G	-	-
7	A	JNK1/2/3	T183/Y185, T221/Y223
7	B	AMPKα1	T183
7	C	AMPKα2	T172
7	D	STAT2	Y689
7	E	STAT6	Y641
7	F	PDGF Rβ	Y751
7	G	-	-
8	A	JNK1/2/3	T183/Y185, T221/Y223
8	B	AMPKα1	T183
8	C	AMPKα2	T172
8	D	STAT2	Y689
8	E	STAT6	Y641
8	F	PDGF Rβ	Y751
8	G	-	-
9	A	GSK-3α/β	S21/S9
9	B	Akt1/2/3	S473
9	C	β-Catenin	-
9	D	STAT5α	Y694
9	E	STAT5β	Y699
9	F	STAT5α/β	Y694/Y699

9	G	PBS (Negative control)	-
10	A	GSK-3α/β	S21/S9
10	B	Akt1/2/3	S473
10	C	β-Catenin	-
10	D	STAT5α	Y694
10	E	STAT5β	Y699
10	F	STAT5α/β	Y694/Y699
10	G	PBS (Negative control)	-
**Membrane B**			
**Row**	**Column**	**Target**	**Phosphorylation site**
11	A	-	-
11	B	Akt1/2/3	T308
11	C	p70 S6 Kinase	T389
11	D	p70 S6 Kinase	T421/S424
11	E	STAT3	Y705
11	F	STAT3	S727
11	G	HSP60	-
12	A	-	-
12	B	Akt1/2/3	T308
12	C	p70 S6 Kinase	T389
12	D	p70 S6 Kinase	T421/S424
12	E	STAT3	Y705
12	F	STAT3	S727
12	G	HSP60	-
13	A	p53	S392
13	B	p53	S46
13	C	p53	S15
13	D	RSK1/2/3	S380/S386/S377
13	E	p27	T198
13	F	WNK1	T60
13	G	-	-
14	A	p53	S392
14	B	p53	S46
14	C	p53	S15
14	D	RSK1/2/3	S380/S386/S377
14	E	p27	T198
14	F	WNK1	T60
14	G	-	-
15	A	-	-
15	B	-	-
15	C	c-Jun	S63
15	D	eNOS	S1177
15	E	PLC-γ1	Y783
15	F	PYK2	Y402
15	G	-	-

16	A	-	-
16	B	-	-
16	C	c-Jun	S63
16	D	eNOS	S1177
16	E	PLC-γ1	Y783
16	F	PYK2	Y402
16	G	-	-
17	A	Reference spot	-
17	B	-	-
17	C	-	-
17	D	-	-
17	E	-	-
17	F	-	-
17	G	PBS (Negative control)	-
18	A	Reference spot	-
18	B	-	-
18	C	-	-
18	D	-	-
18	E	-	-
18	F	-	-
18	G	PBS (Negative control)	-

Figure S2. (a) Results from the phosphokinase array assay in cells treated with aldehyde 13-13. VK2/E6E7 cells were stimulated with 500 µM of aldehyde 13-13 for 30 minutes and phosphorylation intensities normalized to the ethanol-treated control and expressed as a percentage increase or decrease. The x-axis represents the 43 different kinases. The signals spots were analyzed with Image J software. **(b)** Image of the whole blot of the phospho-kinase array. Blue box indicates reference spot, red box indicates AMPKα1. The data table indicates target of each blot and its phosphorylation sites.

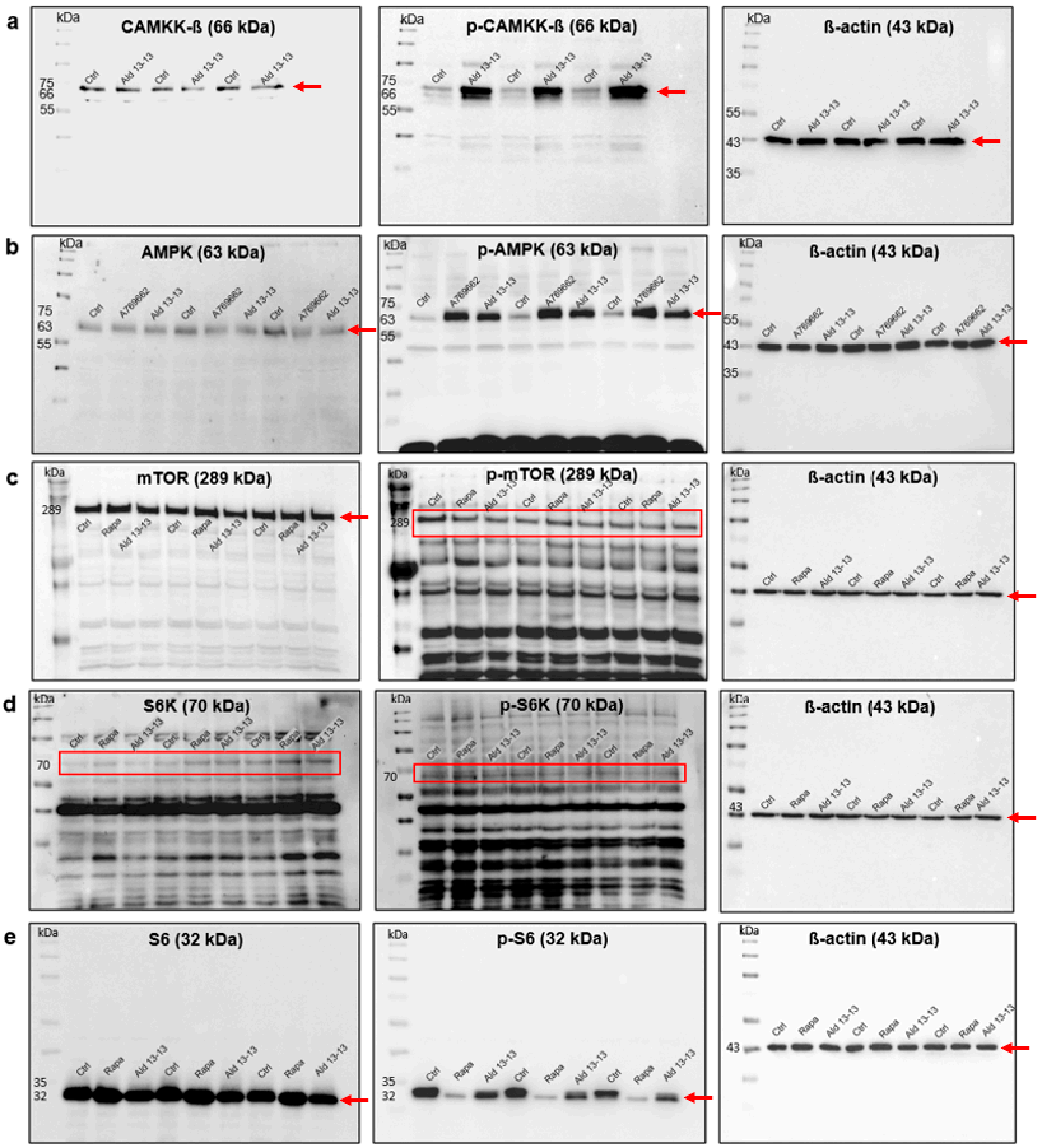

Figure S3. Full length uncropped westernblot images used to generate Fig 4. **(a)** CAMKK-β, p-CAMKK-β and β-actin blot respectively. (**b**) AMPK, p-AMPK and β-actin blot respectively. **(c)** mTOR, p-mTOR and β-actin blot respectively. **(d)** S6K, p-S6K and β-actin blot respectively. **(e)** S6, p-S6 and β-actin blot respectively. Ctrl: control, Ald 13-13: Aldehyde 13-13, A769662: AMPK agonist, Rapa: rapamycin.

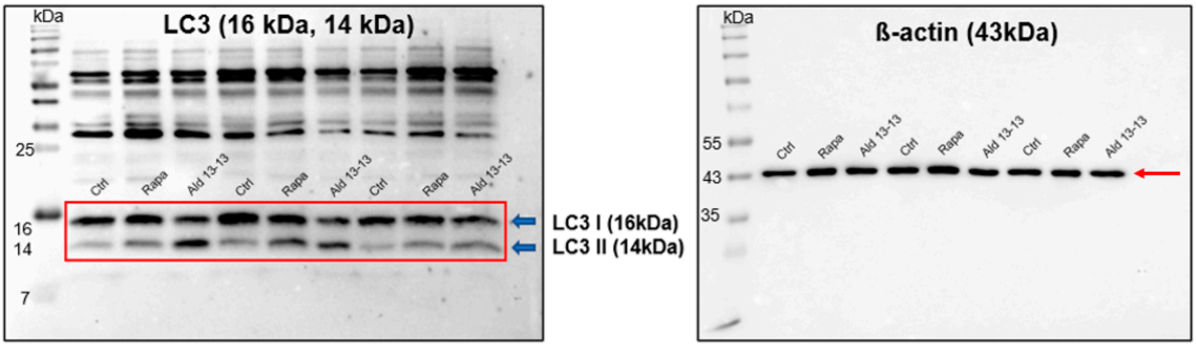

Figure S4. Full length uncropped westernblot images used to generate Fig 5a. LC3 I/II and β-actin blot respectively. Ctrl: control, Rapa: rapamycin, Ald 13-13: Aldehyde 13-13.

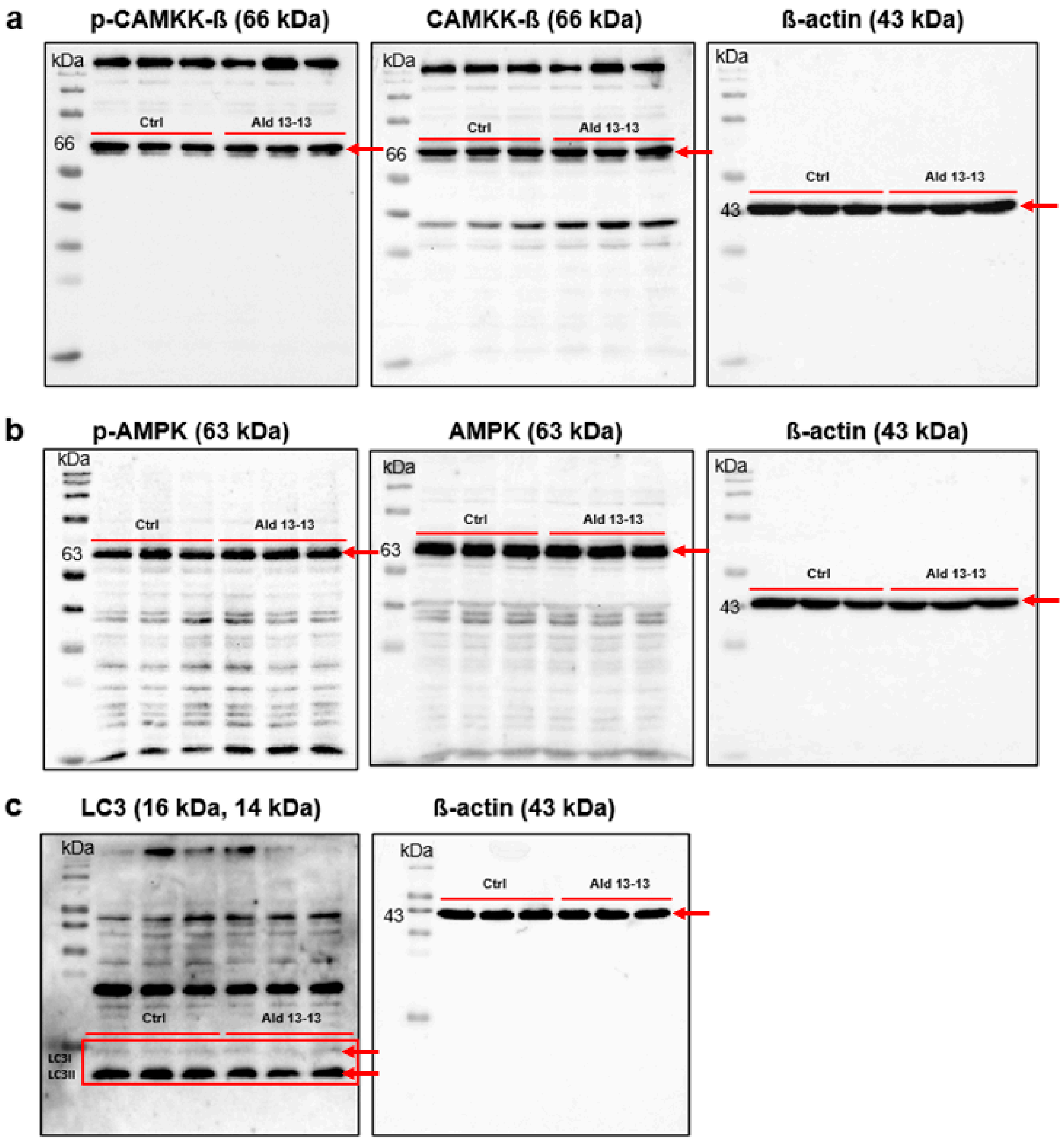

Figure S5. Full length uncropped westernblot images used to generate Fig 6b-d. (**a**) CAMKK-β, p-CAMKK-β and β-actin blot respectively. (**b**) AMPK, p-AMPK and β-actin blot respectively. (**c**) LC3-I/LC3-II and β-actin blot respectively. Ctrl: control, Ald 13-13: Aldehyde 13-13.

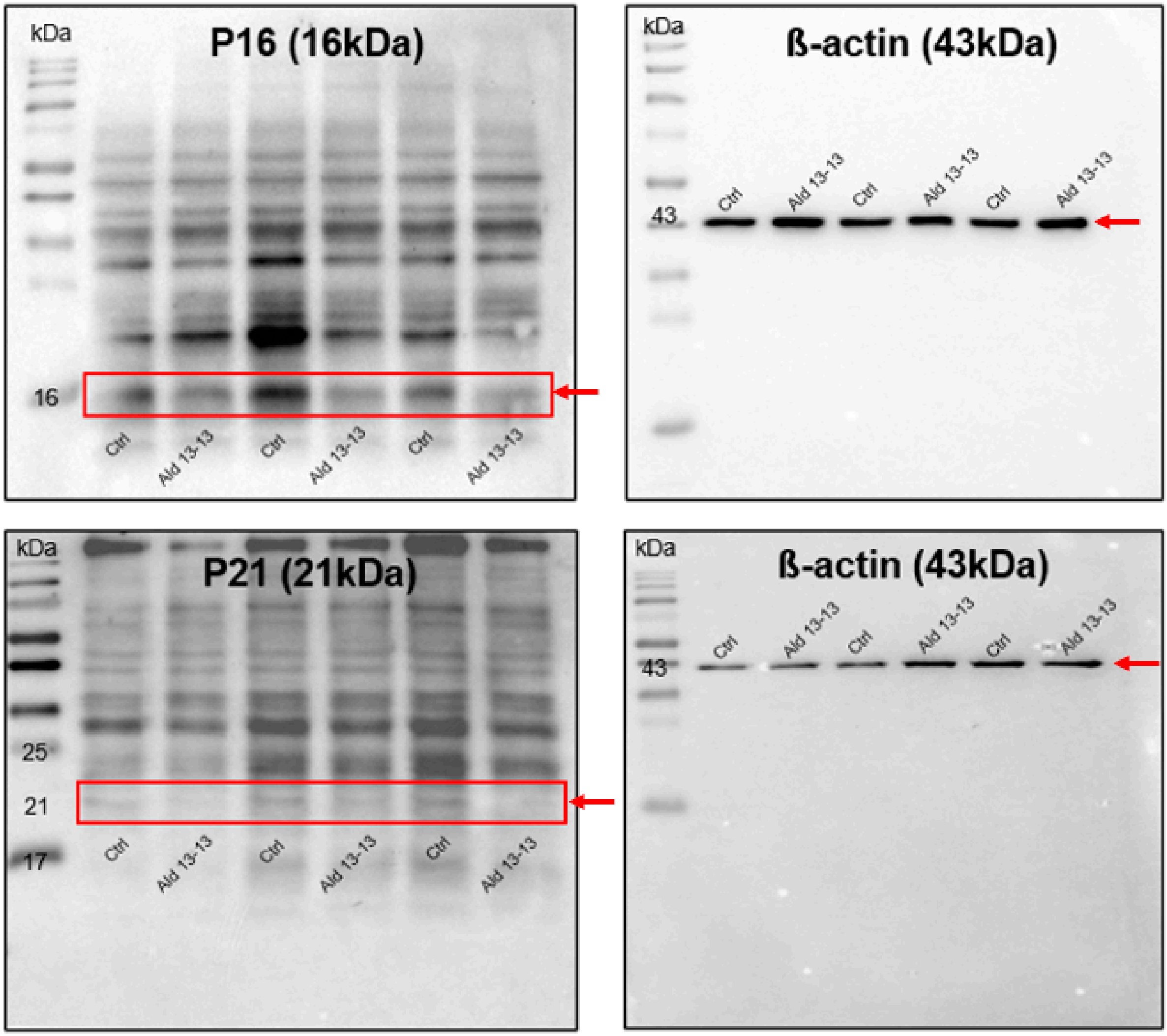

Figure S6. Full length uncropped westernblot images used to generate Fig 7b. P16 and its β-actin blot; P21 and its β-actin blot respectively. Ctrl: control, Ald 13-13: Aldehyde 13-13.

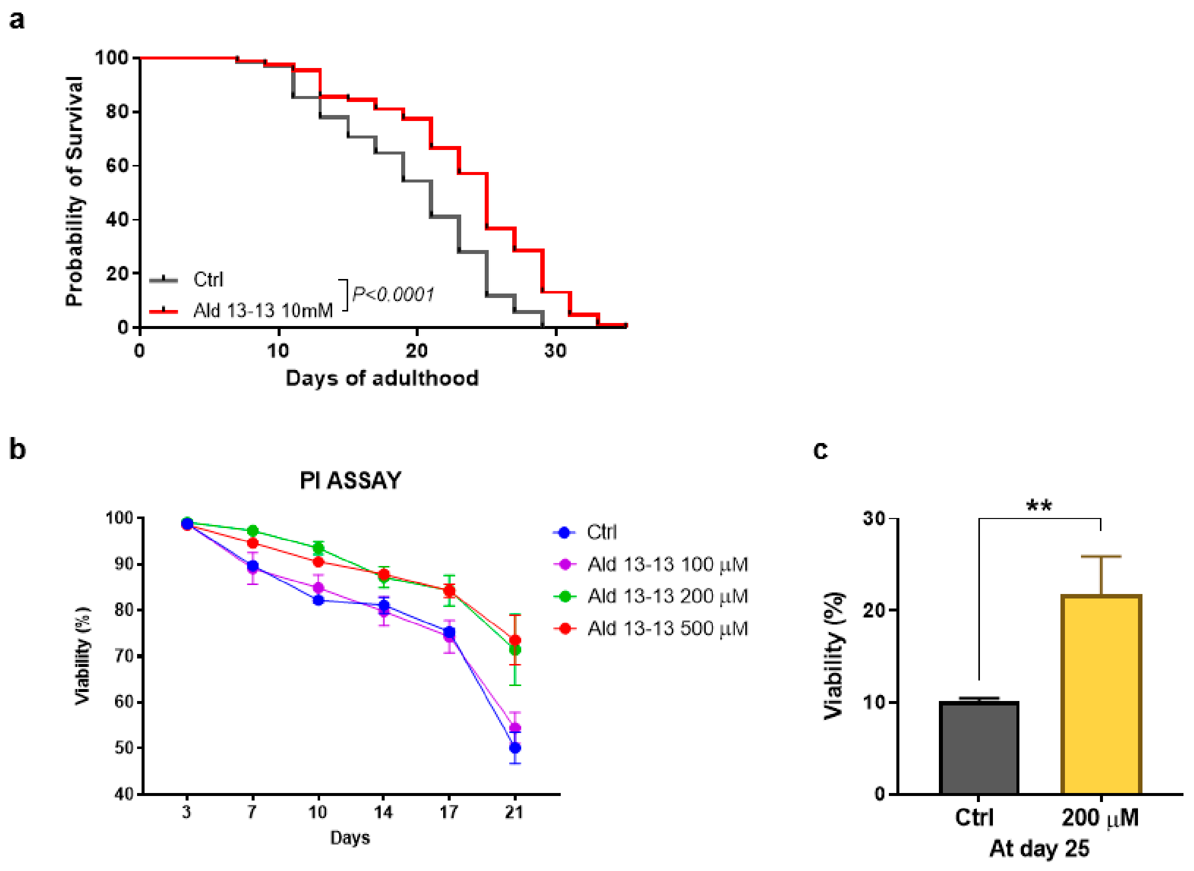

Figure S7. Effect of aldehyde 13-13 on the lifespan of C. elegans and budding yeast. **(a)** Survival of *C. elegans* (black, control; red, aldehyde 13-13 at 10 mM) **(b)** Cell survival assay of budding yeast assessed by propidium iodine (PI) assay (blue, control; purple, 100 µM; green, 200 µM; red, 500 µM of aldehyde 13-13). One-way ANOVA between control and aldehyde 13-13. 100 µM, P = 0.4067; 200 µM, P = 0.0246; 500 µM, P = 0.0275. **(c)** Cell viability of budding yeast at 25 days of experiments. Aldehyde 13-13 (200 µM) was compared to vehicle control. Data are means ± SEM. A two-tailed unpaired *t*-test was performed for multiple or two-group comparisons. *, $P < 0.05$; **, $P < 0.01$; ***, $P < 0.001$.
